# Disruption of the *Toxoplasma gondii* Parasitophorous Vacuole by IFNγ-Inducible Immunity-Related GTPases (IRG Proteins) Triggers Necrotic Cell Death

**DOI:** 10.1371/journal.ppat.1000288

**Published:** 2009-02-06

**Authors:** Yang O. Zhao, Aliaksandr Khaminets, Julia P. Hunn, Jonathan C. Howard

**Affiliations:** Institute for Genetics, University of Cologne, Cologne, Germany; University of Geneva, Switzerland

## Abstract

*Toxoplasma gondii* is a natural intracellular protozoal pathogen of mice and other small mammals. After infection, the parasite replicates freely in many cell types (tachyzoite stage) before undergoing a phase transition and encysting in brain and muscle (bradyzoite stage). In the mouse, early immune resistance to the tachyzoite stage is mediated by the family of interferon-inducible immunity-related GTPases (IRG proteins), but little is known of the nature of this resistance. We reported earlier that IRG proteins accumulate on intracellular vacuoles containing the pathogen, and that the vacuolar membrane subsequently ruptures. In this report, live-cell imaging microscopy has been used to follow this process and its consequences in real time. We show that the rupture of the vacuole is inevitably followed by death of the intracellular parasite, shown by its permeability to cytosolic protein markers. Death of the parasite is followed by the death of the infected cell. The death of the cell has features of pyronecrosis, including membrane permeabilisation and release of the inflammatory protein, HMGB1, but caspase-1 cleavage is not detected. This sequence of events occurs on a large scale only following infection of IFNγ-induced cells with an avirulent strain of *T. gondii*, and is reduced by expression of a dominant negative mutant IRG protein. Cells infected by virulent strains rarely undergo necrosis. We did not find autophagy to play any role in the key steps leading to the death of the parasite. We conclude that IRG proteins resist infection by avirulent *T. gondii* by a novel mechanism involving disruption of the vacuolar membrane, which in turn ultimately leads to the necrotic death of the infected cell.

## Author Summary

*Toxoplasma gondii* infects many warm-blooded animals, including approximately one quarter of the world's human population, residing life-long, usually asymptomatically, in cysts in the brain. If, however, the immune system is weakened for any reason, *T. gondii* can break out and cause life-threatening disease. Furthermore, early *T. gondii* infection can transfer to the fetus and cause damage. Yet, the human is not a natural host for this parasite. *T. gondii* reproduces sexually only in cats and the life cycle depends on carnivory by cats of infected intermediate hosts, normally small mammals like the mouse. If the mouse cannot slow down the infection by immune resistance, *T. gondii* can be lethal. We show that a family of intracellular mouse proteins called immunity-related GTPases (IRGs) can attack *T. gondii* early after it has infected a cell and kill at least some of the parasites. The infected cells die too, but we show that in dying they release a protein that can stimulate local immunity. For most strains of *T. gondii*, this early immune attack is probably enough to prevent the parasite from killing the mouse, and allows the parasite to establish a long-lasting infection in the mouse brain or muscle.

## Introduction

The mouse is a natural intermediate host for *Toxoplasma gondii*, an apicomplexan parasite whose definitive host is the cat. Most *T. gondii* strains are not virulent for normal mice at low infective doses [1],[2]. Following the development of a strongly IFNγ-dependent primary immunity, rapidly replicating tachyzoites convert to the slowly-replicating bradyzoite stage and encyst in brain and muscle without causing severe symptoms, there to await completion of the infection cycle following ingestion by a cat at some later time [3]–[5]. This relatively benign course of infection is, however, drastically altered by disruption of genes encoding key components of the interferon-gamma (IFNγ)-response pathway [6]–[8]. In recent years it has become clear that a group of highly IFN-inducible GTPases, the IRG proteins (formerly p47 GTPases [9]) play an essential role in limiting the early tachyzoite replication stage [10]. Genomic disruption of individual members of this gene family causes normally avirulent *T. gondii* strains to behave as highly virulent pathogens, killing infected mice as early as 10 days after primary infection [11],[12].

The effects of IRG gene disruption on the whole animal are mirrored by the failure of IRG protein-mediated resistance processes occurring in individual *T. gondii*-infected cells. *T. gondii* tachyzoite replication in infected cells can be measured in tissue culture in a variety of cell types, including fibroblasts, macrophages and astrocytes, and pre-treatment of such cells with IFNγ causes potent inhibition [13]–[15]. In cells derived from mice with single disrupted IRG genes, IFNγ-mediated control of *T. gondii* replication is more or less reduced, though rarely completely eliminated [10], [16]–[19]. Marginal loss of control in cells deficient in Irga6 (IIGP1) contrasts with highly significant inhibition in IFNγ-treated wild-type cells expressing a dominant negative form of Irga6 [17], presumably as a result of the high level of interactivity recently documented between the IRG proteins [20]. Up till now, Irgm1 (LRG-47), Irgm3 (IGTP), Irgd (IRG-47), Irga6 and Irgb6 (TGTP) (for the new nomenclature of the p47 GTPases, see reference 50) have all been documented as participating in resistance to *T. gondii* either at the cellular or whole animal levels, or both.

Some years ago we reported the rapid accumulation of IRG proteins on the parasitophorous vacuole membrane (PVM) of *T. gondii* infecting IFNγ-treated mouse astrocytes and the subsequent appearance of vacuoles where the vacuolar membrane was apparently disrupted [17]. We documented local vesiculation and perforation of the IRG-coated PVM, and death of the included parasite as evidenced by penetration of Irga6 into the moribund parasite detected by immunoelectronmicroscopy. These findings have been confirmed in more recent studies both in macrophages [19],[21] and very recently again in astrocytes [16]. In the present report we have used live cell imaging to be able to put a time scale on these events. We are able to document the disruption of IRG protein-loaded vacuoles within the first hours after infection. Approximately 20 minutes after disruption of the vacuole the parasite itself is dead, as documented by its permeability to cytosolic protein markers. About an hour after the death of the parasite the infected cell itself undergoes necrotic death, losing plasma membrane integrity and releasing the chromatin modelling protein, HMGB1, known to be a potent pro-inflammatory stimulus [22]. We show in two ways that cellular necrotic death depends on the disruption of the PVM: firstly, by direct observation, cellular necrosis occurred only after the disruption of at least one PVM and, secondly, both vacuolar disruption and cellular necrosis occur very rarely in fibroblasts infected with virulent strains of *T. gondii*. Our results thus connect the action of IRG proteins at the PVM directly to mouse resistance to *T. gondii* at both the cellular and whole animal levels.

## Results

### Disruption of IRG-positive *T. gondii* vacuoles observed in live-cell microscopy

**Figure 1:**
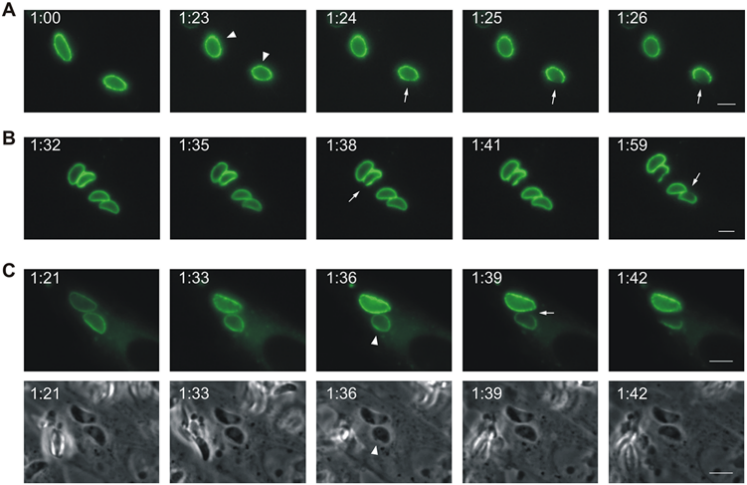
Disruption of IRG-positive *T. gondii* vacuoles. MEFs were transfected with an expression plasmid encoding Irga6-ctag1-EGFP and treated with 200 U/ml IFNγ. 24 hours later cells were infected with ME49 strain *T. gondii* at an MOI between 5 and 10 for 1 hour and observed microscopically by time-lapse photography. Images were taken at 1 minute (A and B) or 3 minute (C) intervals. In C, both fluorescence and corresponding phase contrast images are shown. Selected images from the series show the disruption of Irga6-ctag1-EGFP positive *T. gondii* vacuoles. The number of PVs in individual cells were 3, 4 and 2 for A, B and C, respectively. Disruption of IRG-positive PVM is indicated by arrows. It was frequently observed that vacuoles and the included parasites tended to round up shortly before disruption (A and C: arrowheads). The timing is indicated as hour:minute post infection. Scale bar: 5 µm. The videos from which these frames were extracted are presented as Video S1(Fig. 1A), S2(Fig. 1B), and S5 and S6(Fig. 1C).

We reported the accumulation of several IRG proteins at the PVM in *T. gondii*-infected, IFNγ-stimulated cells [17]. To monitor subsequent events at the PVM in live cell microscopy, Irga6 was tagged at the C-terminus with EGFP. A short linker sequence, ctag1, between the Irga6 coding region and the EGFP was also required to prevent aggregation of the tagged protein *in vivo* (see Materials and Methods). The correctly tagged Irga6 protein, Irga6-ctag1-EGFP, localises to the endoplasmic reticulum like wild-type Irga6 in IFNγ-induced cells ([17] and unpublished) and accumulates on the PVM of *T. gondii*-infected, IFNγ-induced cells. Irga6-ctag1-EGFP was transfected into IFNγ-induced mouse embryonic fibroblasts (MEFs) that were then infected with *T. gondii* ME49 strain. Vacuoles with Irga6-ctag1-EGFP accumulations were followed in live-cell microscopy. Vacuolar rupture was seen as a sudden breach at a single point in the normally smooth ring of Irga6-ctag1-EGFP surrounding the PV (Fig. 1 and Videos S1, S2, S5). In fixed cell microscopy we have previously shown that such breaches also correspond to breaches in the GRA7 signal at the PVM, confirming that they correspond to breaches in the parasitophorous vacuole membrane (see panel B of the fifth figure in [17]). Over a period of about 5 minutes the breach widened, usually continuing until the visible Irga6 was accumulated at one pole of the organism (see Fig. 1 and see also panel E of the second figure in [17]). It was frequently observed that the generally banana-shaped form of the PVM that follows the shape of the included parasite became rounded up shortly before rupture (Fig. 1a, c, white arrowheads). After rupture of the PVM the parasite reverted to its banana shape (compare the phase contrast images at 1 hour 36 min after addition of *T. gondii* (1:36, see Materials and Methods), before vacuolar disruption, and 1:42, after vacuolar disruption). The rounding up, the sudden development of the rupture, the rapid expansion of the breach and the reversion of the parasite to its banana shape after PVM rupture all suggest that the PVM is put under tension before it ruptures. The live cell observations reported here correspond generally to those very recently reported in astrocytes using Irgm3-EGFP (IGTP) instead of Irga6-ctag1-EGFP as a monitor of *T. gondii* vacuolar rupture [16].

### *T. gondii* become permeable to cytosolic proteins after vacuolar rupture

**Figure 2:**
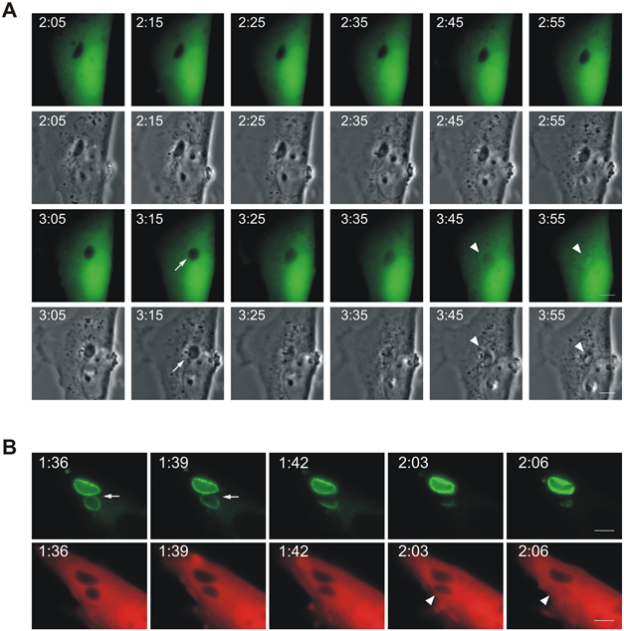
*T. gondii* is permeabilised in the host cytosol after the disruption of PVM. (A) MEFs were induced with 200 U/ml IFNγ and transfected with pEGFP. 24 hours later they were infected with ME49 strain *T. gondii* at an MOI between 5 and 10 for 2 hours and observed microscopically by time-lapse photography. One intracellular parasite was observed throughout the experiment. Images were taken at 5 minute intervals, and selected images from the series (EGFP and phase contrast) are shown. The intracellular *T. gondii*, initially seen as a dark form excluding EGFP, became permeable to EGFP between 3:45 and 3:55 after infection (arrowheads). At the same time there was a detectable change in the appearance of the parasite in the phase contrast series from dense to light at 3:25 that probably corresponded to the rupture of the vacuole. Arrows indicate the rounding up of *T. gondii* also noted in Fig. 1A and C. (B) MEFs were treated with IFNγ and co-transfected with Irga6-ctag1-EGFP and pCherry for 24 hours. Cells were then infected with ME49 strain *T. gondii* for 1 hour and observed by time-lapse microscopy. Two PVs were observed within this cell. Images were taken at 3 minute intervals, and selected images from the series are shown (upper panel Irga6-ctag1-EGFP and lower panel Cherry). The *T. gondii* PVM was disrupted at 1:39 (arrows) and the parasite was permeabilised at 2:06 (arrowheads). These images partially overlap with and extend the series shown in Fig. 1C. Scale bar: 5 µm. The videos from which these frames were extracted are presented as Videos S3 and S4(Fig. 2A) and S5, S6, S7, and S8(Fig. 2B).

In our earlier report we observed by immunoelectronmicroscopy that the normally partially cytosolic Irga6 was frequently found inside moribund *T. gondii* [17], suggesting that dying or dead intracellular *T. gondii* in IFNγ-induced cells become permeable to cytosolic proteins. To examine this further, we used modified GFP proteins, EGFP and Cherry, as markers for the cytosolic pool while observing individual *T. gondii* vacuoles by live cell imaging (Fig. 2 and Videos S3, S4, S7, S8). In Fig. 2A, vacuoles containing impermeable *T. gondii* are seen as dark forms excluding EGFP. During the observation period individual vacuoles suddenly fill with EGFP (Fig. 2A and Videos S3 and S4). The complete disappearance of the *T. gondii* “shadow” implied not only that the PVM became suddenly permeable to soluble fluorescent protein, but also that the parasite itself, still visible in the phase-contrast images, became permeable. Thus at this point the parasites are clearly dead. Monitoring the influx of a cytosolic protein marker, while efficient and sensitive, did not tell us when the vacuole ruptured relative to the moment of permeabilisation of the parasite. Is the PVM permeable to proteins, and the parasite dead, before the vacuolar membrane is visibly ruptured? Or does rupture of the PVM coincide with entry of the fluid phase marker, allowing for the possibility that the parasite is already dead when the PVM ruptures, or perhaps that permeabilisation of the parasite is essentially simultaneous with the rupture of the PVM? Finally, there was the possibility that PVM rupture significantly precedes permeabilisation of the parasite, perhaps implying that the rupture of the PVM is a precondition for the death of the parasite as monitored by breakdown of the permeability barrier. To resolve this issue we turned to two-color live cell imaging, loading IFNγ-induced cells by transfection simultaneously with Irga6-ctag1-EGFP and soluble Cherry before infection (Fig. 2B and Videos S5, S6, S7, and S8). With this approach we were able to show conclusively that the PVM ruptures significantly before the permeabilisation of the included parasite. In Fig. 2B the PVM disrupts at 1:39 and the *T. gondii* becomes permeable to Cherry at 2:06, thus 27 minutes after visible disruption of the PVM. In several observations with this double-labelling technique we could conclude that all *T. gondii* contained in ruptured vacuoles are dead within 20–40 minutes after rupture. Thus rupture of the PVM leads inexorably to the death of the included parasite. Naturally it is likely that the parasite is irretrievably committed to die before the membrane permeability barrier breaks down, but we have no convenient assay for earlier events.

### Dominant-negative Irgb6 inhibits the killing of *T. gondii* in IFNγ-induced fibroblasts

**Figure 3:**
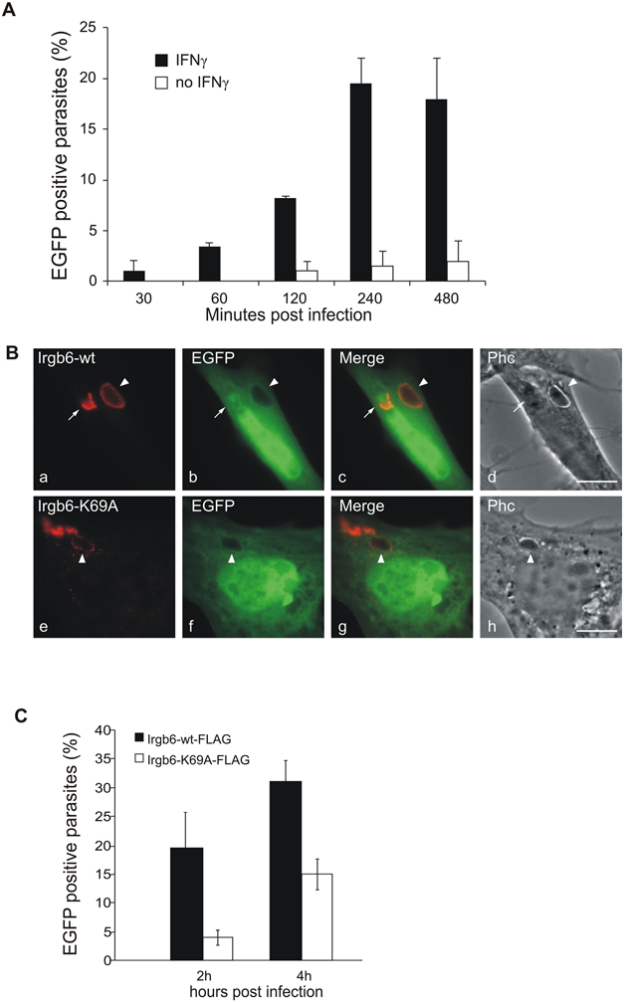
Irgb6 contributes to the IFNγ-dependent killing of *T. gondii*. (A) MEFs were treated with 200 U/ml IFNγ (black bar) or left untreated (white bar) and transfected with pEGFP. After 24 hours cells were infected with ME49 strain *T. gondii* at MOI between 5 and 10 for the indicated times, fixed and stained for GRA7 to identify intracellular *T. gondii*. Permeabilised *T. gondii* containing EGFP were counted and are shown as the percentage of the total number of intracellular parasites counted in EGFP-transfected cells at each time point. Means and ranges of values from two experiments are shown. (B) MEFs were treated with 200 U/ml IFNγ and co-transfected with pEGFP and expression plasmids encoding Irgb6-wt-FLAG (a–d) or Irgb6-K69A-FLAG (e–h). 4 hours after infection with ME49 strain *T. gondii* at an MOI between 5 and 10, cells were fixed and stained for FLAG tag (red). Note the typical cytoplasmic aggregates of Irgb6-K69A-FLAG not associated with the *T. gondii* PV, and the very weak staining of the PVM itself, as documented elsewhere [20]. Arrows indicate a permeabilised *T. gondii* in a disrupted vacuole while the arrowheads indicate EGFP-impermeable parasites in intact vacuoles. Scale bar 10 µm. (C) Quantification of permeabilised *T. gondii* at 2 and 4 hours after infection as described in (B). Means and ranges of values from two experiments are shown. 100–200 PVs per data point were counted blind.

A role of IRG proteins in this sequence of events is suggested but not formally demonstrated by these experiments. IRG proteins have however been shown to be required for successful IFNγ-dependent control of *T. gondii* replication, both by the relaxation of this restriction documented in cells with deleted Irgm1 or Irgm3 genes [10],[16],[18] and by similar loss of control following transfection of dominant negative Irga6 [17] and Irgb6 (unpublished) mutants. We now wished to show that permeabilisation and therefore death of *T. gondii* in IFNγ-induced cells was also dependent on IRG proteins. We therefore adapted the permeabilisation assay demonstrated in Fig. 2 to fixed cells in order to be able to obtain quantitatively significant data. MEFs were transfected with the pEGFP expression plasmid, infected with *T. gondii*, fixed at different times after infection then stained with antibodies against the *T. gondii* dense granule protein, GRA7, which is expressed in the PVM. Cells were examined by conventional fluorescence microscopy for disrupted vacuoles containing permeabilised *T. gondii*. Fig. 3A shows that permeabilised *T. gondii* were first found 30 minutes after infection, their numbers rising continuously to a plateau of about 20% of all vacuoles. In uninduced cells, a low percentage of permeabilised parasites was also seen after 2 hours, perhaps attributable to a low level of stimulation by Type I IFN released from primary MEFs as a result of the transfection procedure. This assay was then used to count permeabilised parasites in IFNγ-induced cells transfected with either wild-type Irgb6 or its dominant negative mutant, Irgb6-K69A, both FLAG-tagged at the C-terminus. Irgb6-K69A, like the homologous mutant of Irga6-K82A, can scarcely go to the PVM and forms aggregates in both uninduced and induced cells that trap the wild type IFNγ-induced protein in the cytoplasm and prevent its localisation on the PVM [17],[20]. The Irgb6-K69A protein may be a more efficient dominant negative than Irga6-K82A perhaps because more vacuoles normally load with Irgb6 than with Irga6 (unpublished results). In Fig. 3B (top row) an IFNγ-induced cell transfected with EGFP and wild-type Irgb6-FLAG contains 2 intracellular *T. gondii*. The vacuole to the left (white arrow) is already disrupted as judged by the polar distribution of Irgb6-FLAG (red) and the parasite has obviously already been permeated with EGFP. The vacuole and parasite to the right (white arrowhead) is still intact. In Fig. 3B (bottom row) an IFNγ-induced cell transfected with Irgb6-K69A-FLAG and EGFP contains one intracellular *T. gondii* intact within its vacuole. The PVM is very weakly labelled with Irgb6-K69A-FLAG, which is accumulated elsewhere in aggregates in the cytoplasm. Fig. 3C shows that transfection of the Irgb6-K69A mutant into IFNγ-induced cells causes a reduction in EGFP-positive *T. gondii* at both 2 h and 4 h after infection relative to IFNγ-induced cells transfected with the wild-type Irgb6 construct as a control.

These results support earlier evidence that resistance to *T. gondii* is reduced by dominant negative IRG proteins [17] and indicates that IRG proteins act early after infection.

### Cells containing a ruptured *T. gondii* PVM die by necrosis after the death of the parasite

**Figure 4:**
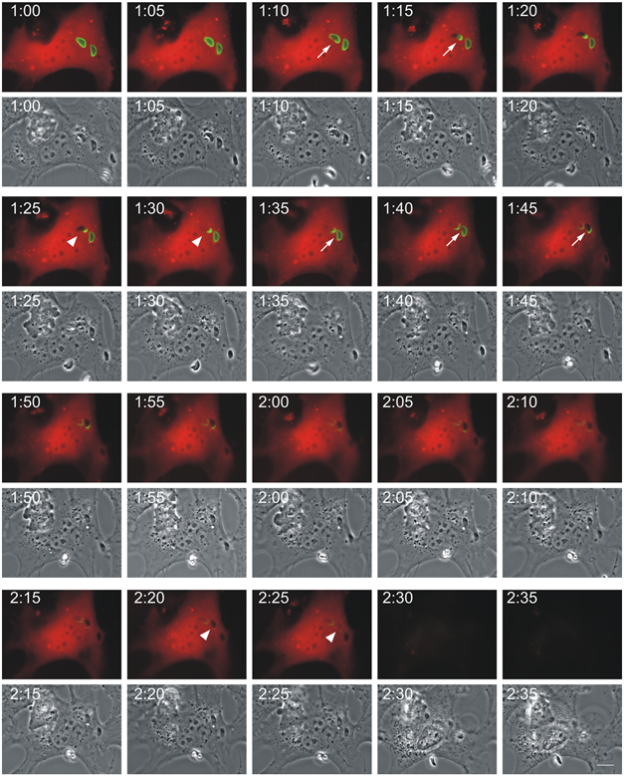
Host cells die after disruption of the PVM and death of the *T. gondii*. MEFs were treated with 200 U/ml IFNγ and co-transfected with Irga6-ctag1-EGFP and pDsRed constructs for 24 hours. Cells were then infected with ME49 strain *T. gondii* at MOI between 5 and 10 for 1 hour and observed microscopically by time-lapse photography. Three PVs were observed within this cell when the experiment started. Images were taken at 5 minutes intervals, and the complete series is shown. Two Irga6-ctag1-EGFP-positive PVMs were disrupted at 1:10 and 1:40, respectively (arrows). These two *T. gondii* became permeable to mDsRed at 1:30 and 2:25, respectively (arrowheads). At 2:30 after infection, the mDsRed signal suddenly disappeared from the host cell accompanied by a drastic change in host cell morphology shown in phase contrast images. Scale bar: 10 µm. The videos from which these frames were extracted are presented as Videos S9 and S10.

We observed in live cell imaging that the fluid phase fluorescent protein markers suddenly and invariably disappeared from the cell shortly after the permeabilisation of the parasite. In Fig. 4 (see also Videos S9 and S10) is shown the complete sequence of events in an IFNγ-induced MEF expressing Irga6-ctag1-EGFP and mDsRed, infected with 3 *T. gondii* parasites, two with conspicuous Irga6-ctag1-EGFP positive PVMs. The upper ringed vacuole disrupts at 1:10 and the parasite becomes permeable between 1:25 and 1:30. The lower ringed vacuole disrupts at 1:40 and becomes permeable between 2:20 and 2:25. At 2:30 the cytosolic mDsRed is suddenly lost from the cell, one hour and 20 minutes after the disruption of the first vacuole. Parallel phase contrast images of the film sequences showed a drastic restructuring of the cytoplasm coinciding with the disappearance of the cytosolic marker at 2:30. This crisis clearly corresponded to the death of the cells. Thus the nucleus suddenly condensed and was separated from the cytoplasm by a clear phase-dense margin, and the cell ceased active movement. There was no sign of either nuclear or cytoplasmic blebbing. We never observed similar effects occurring in neighbouring uninfected cells, nor in infected cells in which neither PVM rupture nor parasite death had occurred. In repeated observations, loss of cell membrane integrity regularly occurred something over an hour after disruption of a PV. The death of the infected cell, like the permeabilisation and death of the parasite, seems also to be an inexorable consequence of a sequence of physiological events beginning with the rupture of the PVM.

**Figure 5:**
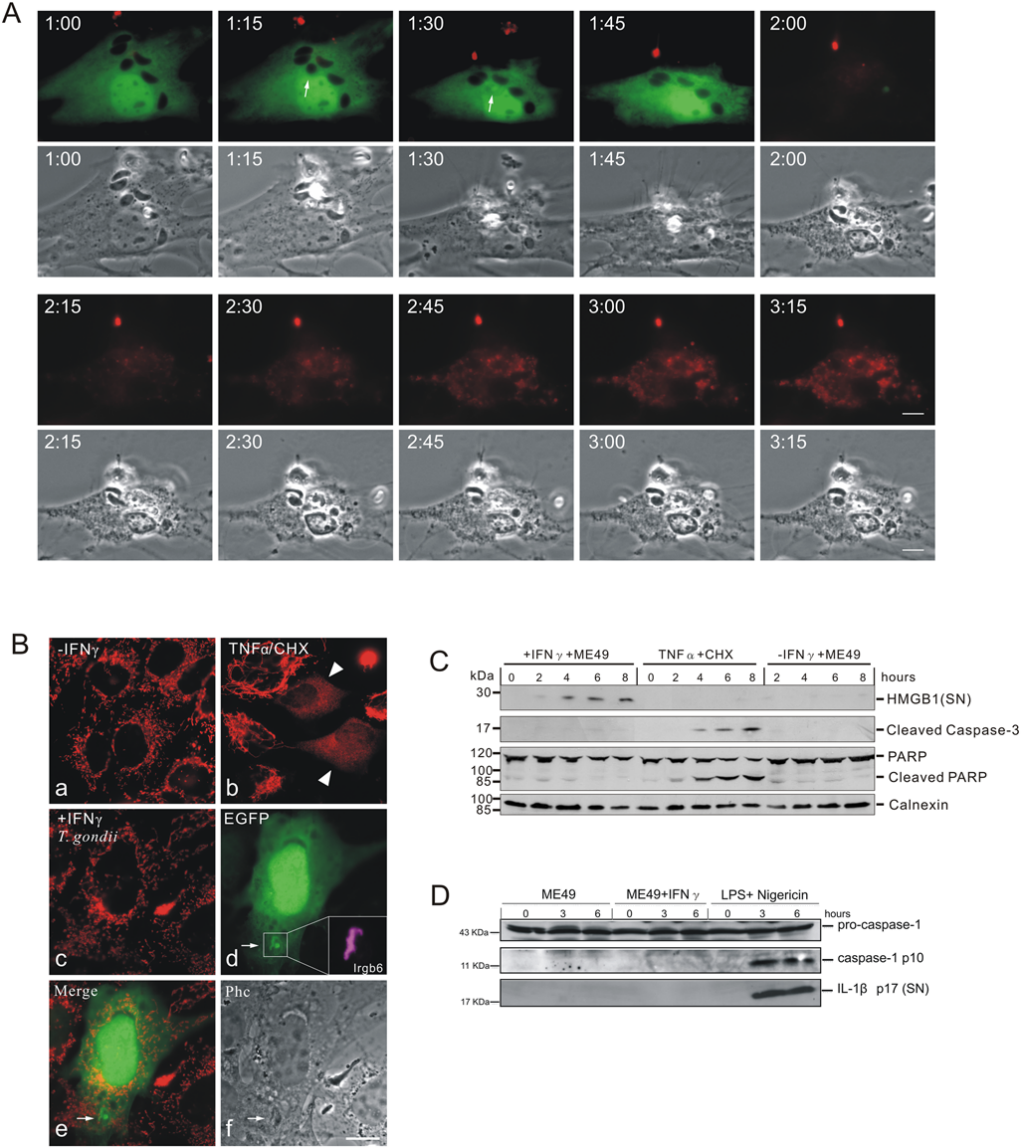
IFNγ-treated *T. gondii* infected host cells undergo necrosis after vacuolar disruption. (A) Phosphatidylserine is not expressed on the cell surface before death. MEFs were induced with 200 U/ml IFNγ and transfected with pEGFP. After 24 hours they were infected with ME49 strain *T. gondii* for 1 hour at MOI between 5 and 10 and observed microscopically by time-lapse photography. Phosphatidylserine was detected by adding 1% (v/v) Alexa-555-labeled annexin V with 2.5 mM CaCl_2_ into the medium. Images were taken at 5 minute intervals and selected images from the series are shown. One intracellular *T. gondii* rounded up and was permeabilised at 1:30 (arrows) and the host cell died at 2:00. No annexin V signal was detected on the plasma membrane before cell death. After permeabilisation of the plasma membrane, annexin V accumulated steadily on intracellular material. Scale bar: 10 µm. The videos from which these frames were extracted are presented as Videos S11 and S12. (B) Cytochrome C is not released from mitochondria in cells containing a disrupted vacuole. MEFs were induced with 200 U/ml IFNγ and transfected with pEGFP for 24 hours (c–f). Cells were then infected with ME49 strain *T. gondii* at MOI between 5 and 10 for 4 hours, fixed and stained for cytochrome C (red) and Irgb6 (inbox in d). Arrows indicate the permeabilised *T. gondii* and Irgb6 staining shows the disrupted PVM. As control, MEFs without any treatment (a), or treated with TNFα (40 ng/ml) and cycloheximide (10 µg/ml) for 4 hours to induce apoptosis (b), were stained for cytochrome C. Arrowheads indicate cells showing release of mitochondrial cytochrome C. Scale bar 10 µm. (C) HMGB1 is released from IFNγ-induced cells infected with *T. gondii*. MEFs were treated with 200 U/ml IFNγ or left untreated for 24 hours and infected with ME49 *T. gondii* for the indicated times. Cells were then lysed and the lysates blotted for cleaved caspase-3 and PARP, as well as calnexin as loading control. Cell culture supernatants were collected at the indicated times after infection and blotted for HMGB1. MEFs treated with TNFα (40 ng/ml) and cycloheximide (10 µg/ml) were blotted as control for apoptotic cells. (D) Caspase-1 and IL-1β are not activated. BMMs were treated as described in (C). Cell lysates were blotted for caspase-1 and cell culture supernatant were blotted for IL-1β. BMMs were first treated with LPS (1 µg/µl) for 24 hours, and then treated with nigericin (20 µM) for the indicated times to activate inflammasomes as positive control.

The apparently reactive death of the parasite-infected, IFNγ-induced cells with sudden permeabilisation of the cell membrane was suggestive of necrotic rather than apoptotic death [23]. Consistent with this, when IFNγ-induced, EGFP-transfected cells were infected with *T. gondii* and incubated with soluble Alexa-555-labeled annexin V to detect phosphatidyl serine [24], the apoptotic marker was at no time detectable on the plasma membrane (Fig. 5A and Videos S11 and S12). After loss of EGFP, annexin V accumulated slowly on internal membranes. We next examined loss of cytochrome C from mitochondria as an early indication of apoptosis [25], using immunofluorescence on fixed IFNγ-induced cells transfected with EGFP as a fluid phase marker and infected with *T. gondii* (Fig. 5B). In no cell containing a permeabilised *T. gondii*, and therefore due to die within an hour or so, did we see release of cytochrome C into the cytoplasm (Fig. 5B panels c–f). When apoptosis was actively induced by treatment of the cells with TNFα and cycloheximide [25], cytoplasmic cytochrome C was present in many of the treated cells (Fig. 5B panels a, b). Furthermore, no cleavage of caspase-3 could be detected in western blots of lysates of IFNγ-induced, *T. gondii*-infected cells up to 8 hours after infection, nor cleavage of the caspase-3 substrate PARP, both of which signs of apoptosis [26] were easily detected in MEFs treated with cycloheximide and TNFα [25] (Fig. 5C). On the other hand, the pro-inflammatory chromatin binding protein, HMGB1 [22], was detected as early as 2 h after infection in the supernatant of the IFNγ-induced, *T. gondii* infected cells but not in the supernatants of the cycloheximide and TNFα-treated cells (Fig. 5C).

From these results we could conclude that disruption of the PVM and death of the included *T. gondii* initiates a non-apoptotic death in IFNγ-induced cells with some features of necrosis. By analogy with other pathogen-induced necrotic processes [27]–[29], we considered it likely that activation of caspase-1 by the inflammasome would be found [30]. However we were unable to demonstrate activation of caspase-1 or its substrate IL-1β in IFNγ-induced positive control MEFs stimulated by LPS and treatment with nigericin to induce necrosis [31]. We therefore turned to primary bone marrow derived macrophages (BMMs), which respond to IFNγ induction and *T. gondii* in the same way as MEFs, ending in cell death (unpublished results, and see below). Neither cleavage of caspase-1 nor processing of IL-1β were detected in IFNγ-induced BMMs infected with *T. gondii* for 6 hours compared with cells treated with LPS and nigericin as a positive control for inflammasome-mediated necrotic death [32] (Fig. 5D). This suggests that the IFNγ-dependent necrosis seen in *T. gondii*-infected cells may be related to the pyronecrosis reported in mouse macrophages infected with *Shigella flexneri* where caspase-1 cleavage was shown not to be required for necrosis [33].

The necrotic death of infected cells about an hour after the permeabilisation of the parasite explains why the peak percentage of permeabilised *T. gondii* cannot rise above about 20% (Fig. 3A). When the cell dies by necrosis it detaches and is lost during the processing of slides. Thus the only live cells that can be detected containing a permeabilised vacuole are those seen in the approximately 1 hour long window between parasite permeabilisation and cell death.

In view of the apparent inevitability of the post-vacuolar disruption-dependent necrotic death, it was surprising that this effect has not been reported before. To illustrate the scale of the phenomenon, we prepared a simple overview image of IFNγ-induced fibroblasts infected 8 hours previously with T. gondii ME49 strain at a MOI of 5 and washed free of floating T. gondii at 2 hours after infection. Propidium iodide was added immediately before microscopic examination of the living culture. Strikingly large numbers of heavily condensed, propidium iodide stained nuclei were seen, indicating dead cells, compared with infected control cells not induced with IFNγ (Fig S1).

### Macroautophagy is not involved in the disruption of the PVM and death of *T. gondii* in IFNγ-induced cells

**Figure 6:**
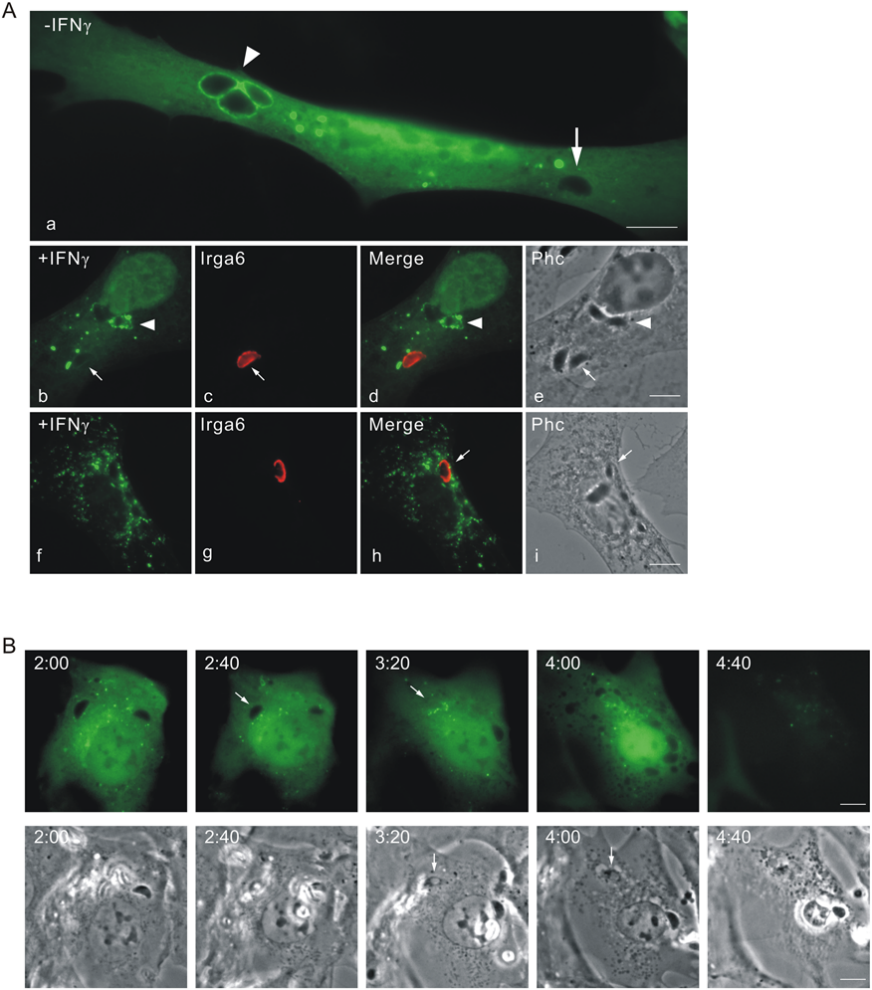
Macroautophagy is not involved in the disruption of the *T. gondii* PVM or the death of the parasite. (A) MEFs were left uninduced (a) or induced with 200 U/ml IFNγ (b–i) and transfected with pEGFP-LC3. After 24 hours cells were infected with ME49 strain *T. gondii* at an MOI between 5 and 10 for 6 hours. Cells were then fixed and stained for Irga6 (red, b–i). Arrows indicate the Irga6-positive PVM and arrowheads indicate EGFP-LC3-associated *T. gondii* vacuoles. Scale bar 10 µm. (B) MEFs were induced with 200 U/ml IFNγ and transfected with pEGFP-LC3. After 24 hours cells were infected with ME49 strain *T. gondii* at an MOI between 5 and 10 for 2 hour and observed microscopically by time-lapse photography. Two PVs were observed within this cell. Images were taken at 5 minutes intervals, and selected images from the series are shown. One *T. gondii* was obviously permeabilised and incorporated free cytosolic EGFP-LC3 at 3:00 after infection and the host cell died at 4:35 after infection. There is no clear association between EGFP-LC3 and *T. gondii* vacuoles. Scale bar 10 µm. The videos from which these frames were extracted are presented as Videos S13 and S14.

Following recent evidence for the control of certain bacterial infections by engulfment of the microbes in autophagic membranes [34]–[36], Yap and colleagues employed electron microscopical evidence to implicate autophagy in the destruction of *T. gondii* in *in vivo*-activated mouse peritoneal macrophages [19]. We had earlier reported the presence of EGFP-LC3 vesicular structures in the vicinity of some disrupted *T. gondii* vacuoles in IFNγ-induced astrocytes, though no clear-cut co-localisation could be observed [17]. To re-examine this issue we again observed the behaviour of EGFP-LC3, both in fixed cell (Fig. 6A) and live-cell imaging (Fig. 6B and Videos S13 and S14) in MEFs infected with *T. gondii* ME49 strain. In the absence of IFNγ induction we occasionally saw some EGFP-LC3 associated with *T. gondii* vacuoles. Fig. 6A, top panel, shows a very rare cell with three *T. gondii* vacuoles with LC3 concentrated at the vacuoles (white arrowhead); a fourth vacuole in the same cell (white arrow) is not ringed with EGFP-LC3, and there is no sign of general formation of LC3-positive punctae. When the cells were previously induced with IFNγ, EGFP-LC3-positive structures could be found associated with occasional vacuoles, independently of whether Irga6 positive or negative (Fig. 6A, middle and lower panels). Equally, vacuoles with clearly disrupted PVMs (Fig. 6A, lower panel) were no more likely to be EGFP-LC3 positive than intact vacuoles (Fig. 6A, middle panel). EGFP-LC3-positive punctae were sometimes increased at later times after infection although at no time was there any obvious correlation between LC3 signals and Irga6-positive vacuoles. Indeed the majority of vacuoles in IFNγ-induced cells showed no association with LC3-positive structures.

In view of the heterogeneity of association of EGFP-LC3 with vacuoles in fixed-cell preparations we chose to examine the dynamic behaviour of the autophagic marker in live-cell imaging. Fig. 6B shows consecutive frames of an IFNγ-induced MEF infected with ME49 *T. gondii*. The EGFP-LC3 signal is uniformly distributed in the cell with some punctae, which show no striking association with the two visible *T. gondii* vacuoles. Between frames 2:40 and 3:20 (more accurately in the video between 3:00 and 3:05) after infection (Fig. 6B white arrow) one infecting *T. gondii* becomes permeable to the cytosolic EGFP-LC3 and 95 minutes later the LC3 marker is lost as cell membrane integrity breaks down and the cell undergoes necrotic changes visible in phase contrast (also see Videos S13 and S14). Thus it is clear that the whole series of events from disruption of the vacuole through the death of the *T. gondii* to the necrotic death of the cell can occur without the enclosure of the vacuole in any LC3-positive membranes.

The final elimination of intracellular pathogens through macroautophagy depends on fusion of the pathogen-containing autophagic vacuole with lysosomes followed by degradation of the pathogen [35]. Consistent with the live-cell imaging showing no necessary participation of EGFP-LC3 in the destruction of the pathogen and the initiation of necrosis, no evidence was seen for fusion of *T. gondii*-containing vacuoles with lysosomes, assayed by association with LAMP1, in IFNγ-induced MEFs at any time-point after infection, as we reported earlier [17] and in contrast to the observations of Ling et al [19] (Fig S2). In summary, we found no evidence that macroautophagy plays any necessary role in the IRG-dependent destruction of *T. gondii* in IFNγ-induced MEFs.

### Virulent strains of *T. gondii* do not trigger the necrotic cell death pathway in IFNγ-induced cells

**Figure 7:**
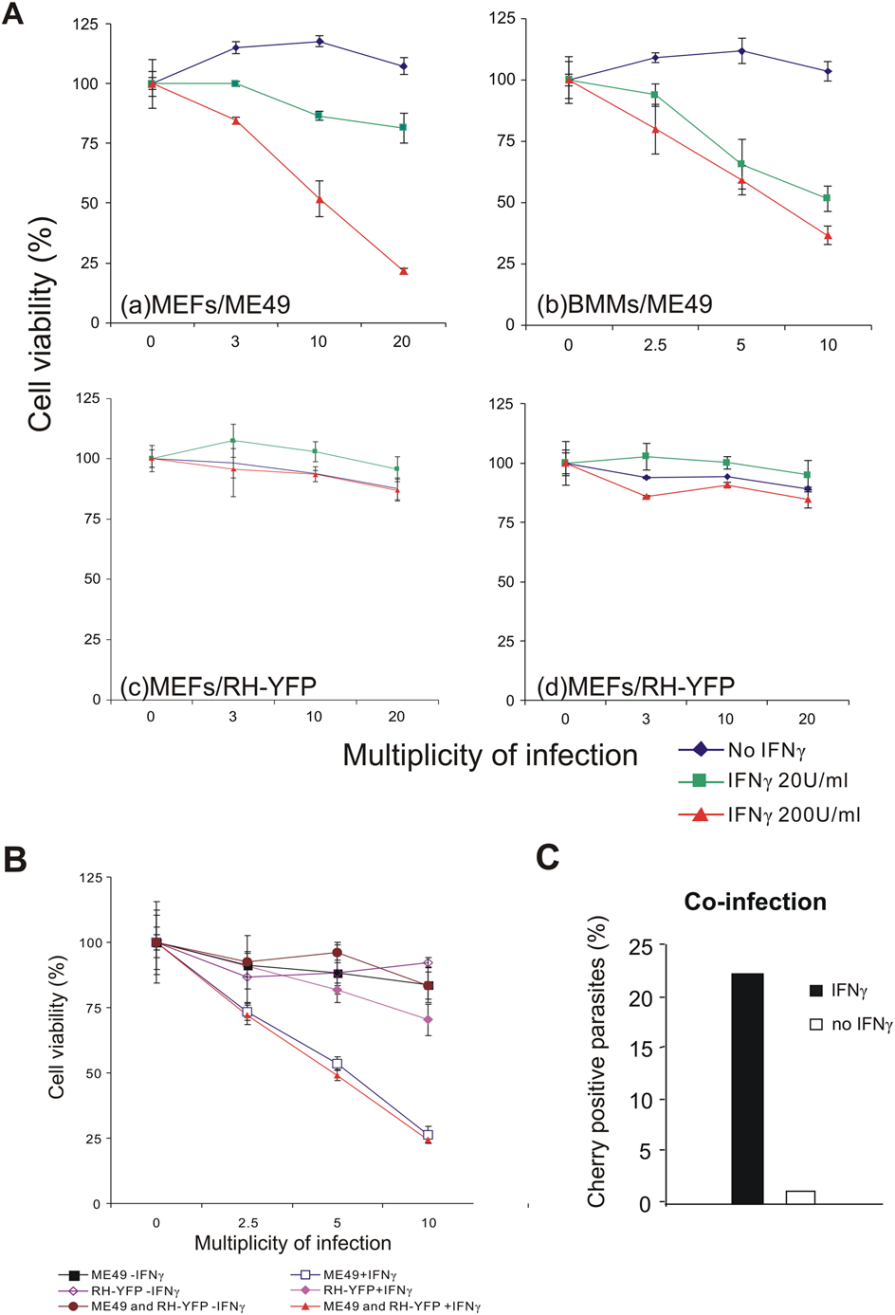
Virulent *T. gondii* are resistant to the IRG- and IFNγ-dependent cell-necrotic programme. (A) MEFs (a, c–d) or BMMs (b) were seeded into 96 well plates and induced with the indicated dose of IFNγ for 24 hours. Cells were then infected with *T. gondii* avirulent strain ME49 (a–b) or virulent strain RH-YFP (c–d) for 8 hours at the indicated MOIs. Cell viabilities were measured and expressed as percentages of those recorded for uninfected cells (MOI = 0). (B) MEFs were treated as described in (A) and infected for 8 hr with ME49 or RH-YFP alone or co-infected with ME49 and RH-YFP simultaneously. Cell viabilities were measured and expressed as percentages of those recorded for uninfected cells. (C) MEFs were transfected with pCherry and induced with 200 U/ml IFNγ for 24 hours (black bar) or left untreated (white bar). Cells were then co-infected with ME49 and RH-YFP *T. gondii* strains (MOI 5 for each strain) for 4 hours. Permeabilised parasites containing Cherry were counted, in cells containing at least one RH-YFP parasite, as a proportion of all intracellular ME49. 200–300 PVs per data point were counted blind.

We have shown elsewhere by ^3^H-uracil incorporation assays that the replication of the virulent type I *T. gondii* strain, RH, is only slightly inhibited by induction of MEFs with IFNγ [37]. We also showed in fixed preparations that IRG proteins failed to accumulate normally on the PVM of infecting RH parasites: fewer vacuoles accumulated each of the IRG proteins tested and the failure of Irgb6 to accumulate on RH vacuoles was especially striking (9% of RH vacuoles positive for Irgb6 compared with 70% of ME49 vacuoles [37]). It was therefore of interest to examine the fate of virulent *T. gondii* in IFNγ-induced cells. The frequency of parasites detected as permeabilised in the fixed cell system exploited above was too low to be estimated above background levels. In view of the temporal correlation between vacuolar loading, vacuolar disruption, *T. gondii* permeabilisation and cellular death by necrosis documented above in cells infected with the ME49 avirulent *T. gondii*, it was of interest to establish whether cells infected with a virulent strain of *T. gondii* also undergo necrotic death. The large scale of necrotic death of IFNγ-induced, *T. gondii* infected cells (Fig S1) can be documented quantitatively at the population level by a standard cell viability assay (Materials and Methods). Fig. 7A shows the results for ME49-infected MEFs (panel a) and BMMs (panel b). Both cell types showed an IFNγ concentration dependent loss of viability with increasing multiplicity of infection. Thus the IFNγ- and *T. gondii*-dependent cell death programme is not distinctive for fibroblasts but, as noted above, also occurs in macrophages. In striking contrast, scarcely any loss of viability was seen in IFNγ-induced MEFs infected with the virulent type I strain RH or its transgenic descendant, RH-YFP, even at the highest MOI (Fig. 7A, panels c, d). However, by direct microscopical observation of IFNγ-induced cells infected with RH strain, very occasional necrotic cells containing apparently moribund *T. gondii* were detected (unpublished). Thus the necrotic process can be initiated by virulent *T. gondii*, but at a far lower frequency. It will be important to establish whether the rare necrotic cells are included in the rare subset of RH-infected cells that load normally with IRG proteins.

This result showed that virulent *T. gondii* are scarcely subject to the IRG-mediated resistance programme. Thus PVMs containing virulent *T. gondii* are generally not disrupted, virulent *T. gondii* are not permeabilised in the cytosol, and the host cells do not undergo necrotic death. In the light of these results, and from the evidence that IRG protein loading of the virulent vacuole is significantly reduced [37], it seemed likely that virulent *T. gondii* prevent the IRG-mediated resistance programme from initiating. To support this conjecture, we examined the behaviour of cells doubly infected with avirulent (ME49) and virulent (RH-YFP) organisms. In the cell viability assay (Fig. 7B) it was clear that doubly-infected MEFs were just as vulnerable to IFNγ- and *T. gondii*-dependent cell death as MEFs infected only with the avirulent strain, showing that the resistance of the virulent strain depends on failure of an earlier event than the necrotic process itself. In co-infected, IFNγ-induced MEFs transfected with Cherry as a fluid phase marker and assayed at 4 hours after infection (Fig. 7C), the presence of the virulent RH-YFP had no impact on the permeabilisation of parasites, which reached the usual figure of over 20% (see Fig. 3A). Thus the resistance of virulent *T. gondii* to IRG proteins is not due to the secretion of a soluble factor that renders the cell incompetent to resist any infecting *T. gondii*, and the vacuolar destruction mechanism is also intact in cells infected with virulent *T. gondii*. We conclude from these results that it is the disruption of the vacuoles that distinguishes avirulent from virulent *T. gondii* strains. Since dominant negative Irga6 and Irgb6 prevent the accumulation of IRG proteins on the PVM [17],[20] and also inhibit vacuole disruption and permeabilisation of the parasite (Fig. 3C), our experiments suggest that the essential difference between virulent type I and avirulent type II *T. gondii* lies in the ability of the virulent parasite to prevent the massive accumulation of IRG proteins on the PVM [37]. The same line of argument leads us also to the conclusion that the critical function of the IRG proteins is probably already fulfilled with the disruption of the PVM.

## Discussion

**Figure 8:**
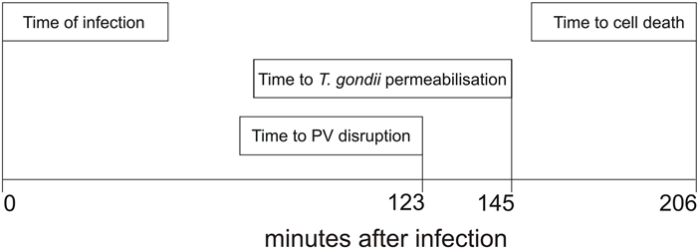
The time-course of IRG-mediated ME49 *T. gondii* resistance. The figure illustrates the “typical” time course for the IRG-mediated programme of resistance against *T. gondii* strain ME49 in IFNγ-induced cells. The values for each step in the programme are the mean values taken from Table S1. It is important to stress that most of these timings are very variable, including the time after adding the *T gondii* to culture at which cell is finally infected. Only the interval between vacuole disruption and *T. gondii* permeabilisation is rather well defined. Nevertheless the data give a correct account of the invariable order of events and their approximate relative timing.

In the present study we have documented a succession of events, beginning shortly after infection of an IFN-stimulated mouse cell by *Toxoplasma gondii*, and ending with the necrotic death of the infected cell. We can characterise this series, as follows: (1) the accumulation of IRG proteins on the parasitophorous vacuole, which begins on some vacuoles as early as 2 minutes after entry of the parasite and typically reaches a maximum between 30 minutes and one hour later (unpublished data), (2) rupture of the IRG-loaded PVM, a process which occurs suddenly and is completed in a few minutes, (3) the permeabilisation of the *T. gondii* plasma membrane documented by entry of fluorescent cytosolic protein markers, which occurs as a sudden event between 20 and 40 minutes after PVM disruption, (4) the necrotic death of the cell, documented by sudden loss of fluorescent cytosolic protein markers and release of the chromatin modelling protein, HMGB1, from the necrotic cell. Integrating our results we can describe an approximate time course for the overall process (Fig. 8 and Table S1). Major variables in this chronology are: the time to initiate PVM loading with IRG proteins, which may be almost immediately following infection to many hours or not at all, for different vacuoles even in the same cell ([17] and unpublished); the time between PVM loading with IRG proteins and vacuole disruption, which can also vary a great deal and may be dependent on the number of loaded vacuoles in the cell (unpublished observations); and lastly the time between permeabilisation of *T. gondii* in a disrupted vacuole and cell death, which has varied from 5 to 105 minutes over our experimental observations. The least variable time interval is that between vacuolar disruption and the permeabilisation of the included *T. gondii*, which, with a single exception at 45 mins has been consistently close to 20 mins.

By several criteria, the fulfilment of this programme is dependent on the activity of IRG proteins. Restriction of *T. gondii* in IFNγ-induced cells has been shown to be dependent on IRG proteins in several earlier studies [10],[17],[18] and two recent studies have specifically shown dependence of vacuolar disruption on the presence of Irgm3 [16],[19]. We show here (Fig. 3) that a dominant-negative mutant of Irgb6 inhibits the appearance of permeabilised *T. gondii* in the IFNγ-induced cell. We also show that cells infected by virulent *T. gondii*, which interfere with PVM loading by IRG proteins ([37] and unpublished), do not undergo necrotic death. As documented extensively elsewhere (Khaminets, unpublished) and confirmed in the present experiments (uncoated *T. gondii* in Fig. 4 and Videos S9 and S10) not all PVM accumulate IRG proteins in multiply infected cells. In our experiments such unloaded vacuoles have never been seen to disrupt. There is thus a clear implication that the accumulation of IRG proteins on a PVM is required for the disruption of that vacuole. Once the vacuolar membrane is disrupted, the rest of the programme moves inexorably forward to the permeabilisation of the parasite and the ultimate necrotic death of the cell. In this scenario, the disruption of the PVM is the critical step that is required for the completion of the programme. We have used permeability to cytosolic proteins as a criterion for death of the parasite because it is unlikely to be controversial. The rather tight linkage in time between vacuolar disruption and permeabilisation of the parasite may suggest that the parasite begins to die at the same time as PVM disruption.

Our experiments do not support the suggestion that the formation of autophagic membranes around disrupted vacuoles [19] plays a necessary role in the unfolding of the programme we describe. LC3-positive membranes undoubtedly form in a proportion of infected cells [17], although in contrast to studies of others based on macrophages [38],[39] we have seen no significant difference between IFNγ-treated and untreated cells (unpublished results). However the full vacuolar destruction and necrosis programme can apparently be implemented without any significant LC3-positive membrane formation (Fig. 6B and Videos S13 and S14). Indeed, the dying parasite exposed in a disrupted vacuole is fully accessible to a cytosolic protein marker, showing that the parasite is not at that stage isolated from the cytosol in an autophagic isolation membrane or membrane-bounded secondary lysosome. This latter conclusion in supported by our failure to find any co-localisation of LAMP1 with the dying parasite (Fig S2). Nevertheless, in an earlier study we observed that transformed fibroblasts from *atg5*-deficient mice were significantly less able to restrict *T. gondii* replication than transformed fibroblasts from control mice of the same strain [13]. In view of the lack of evidence for formation of autophagic membranes at the vacuoles, this result suggests that the effect of atg5 deficiency is not directly connected to the role of atg5 in autophagy. In fact, IFNγ-dependent accumulation of Irga6 on the PVM has recently been shown to be significantly reduced in atg5-deficient primary BMMs [21], a result we can confirm and have extended to other IRG proteins (Khaminets, unpublished). Since the results of the present paper argue that adequate accumulation of IRG proteins on the vacuole is a critical determinant of their function, it seems probable that the reduced control of *T. gondii* replication in atg5-deficient fibroblasts [13] is due to the defect in IRG protein accumulation on the PVM and not to any later effect that can be attributed to deficient autophagy.

The results of this study highlight many unknowns in the cell biology of the relationship between *T. gondii* and its mammalian hosts. We have already stressed that the early loading of IRG proteins onto the PVM is critical for the initiation of the resistance programme, and this is the step that appears to fail with virulent type I parasites [37]. Although loading of the PVM involves transition from the resting GDP-bound to the activated GTP-bound state of some IRG proteins [20],[40], little else is known about the means by which these proteins specifically access the target membrane, nor how this access is impeded by virulent type I *T. gondii*. Is it due to active interference with the IRG proteins, for instance by one of the rhoptry kinases which are known to function as virulence factors [41]–[43], or is the resistance to IRG proteins an intrinsic characteristic of the PV membrane itself, or of the parasite-derived components it contains, due, for example to polymorphism in the structure or presence of an unknown IRG receptor? The disruption of PVs carrying accumulations of active IRG proteins is a striking and now repeatedly confirmed finding [16],[17],[19],[21]. The apparent build-up of tension in the PVM shortly before disruption, seen as a tendency for the vacuole to become spherical, and the speed with which the local disruption spreads strongly suggests that the PVM surface area is being reduced, and this idea would be consistent with EM images showing apparent vesiculation of the PVM [16],[17],[19],[21]. The tendency to view IRG proteins as in some way related to dynamins [44],[45] further fosters the idea that IRG proteins participate in a process of active vesiculation of the PVM. This dynamic model will be hard to test if several IRG proteins participate cooperatively in causing membrane damage [20]. This problem has recently been exacerbated by evidence that members of the family of IFNγ-inducible 65 kDa guanylate binding proteins also target the *T. gondii* PVM and may participate in cell-autonomous resistance [46]. Is this a collaboration with IRG proteins, or a separate enterprise?

Once the PVM disrupts, the fate of the parasite is sealed. In about 20 minutes it becomes permeable to cytosolic proteins but it is unknown both what the cause of death is, and when the process is initiated. From the relatively close temporal correlation between vacuolar disruption and permeabilisation of the *T. gondii* it is plausible that the process is initiated at the time of vacuolar disruption. Why should the cytosol become an inimical environment when the PVM disrupts? Are cytoplasmic immune receptors triggered by material released from the disrupted vacuole? If so, what is the activating material, which are the receptors and what is the mechanism that leads to death of the *T. gondii*? We considered the possibility that reactive oxygen species could contribute to the killing of the parasite, but the entire necrotic programme played out without noticeable deviation from wild-type behaviour in *T. gondii*-infected IFNγ-induced cells derived from p47^phox^-deficient mice (unpublished results) confirming an earlier report that genomic disruption of p47^phox^ does not prevent IFNγ mediated control of *T. gondii in vivo* or *in vitro* [47].

The death of the parasite is followed with the same inevitability by the death of the infected cell. This death is accompanied by no release of mitochondrial cytochrome C, no cleavage of caspase-3 or of its effectors, and no appearance of phosphatidyl serine on the outer leaflet of the plasma membrane, so it is clear that it does not reflect activation of an apoptotic cascade. On the other hand, the dramatic loss of plasma membrane integrity and release of HMBG1 speak strongly for necrosis. The absence of detectable caspase-1 cleavage or release of mature IL-1β recalls the cryopyrin-initiated cell death recently described as pyronecrosis [33] that follows *Shigella flexneri* infection of mouse macrophages, which proceeded unimpaired in infected BMMs from caspase-1 deficient mice. Normal resistance to *T. gondii in vivo* is also reported from caspase-1-deficient mice [48]. There is, however, the important difference that in that study caspase-1 was activated and IL-1β cleaved. Thus if the *T. gondii*-induced necrotic programme indeed proceeds via activation of cryopyrin (NLRP3), the pathway must diverge from the inflammasome before cleavage of caspase-1. Another potentially important difference between the present study and Shigella-induced necrosis is that *T. gondii*-induced necrosis with the properties we describe occurs only in IFNγ-induced cells. While our studies are directed to the role of IRG proteins in driving this process, it is not excluded that further IFNγ-induced components may participate in the terminal necrotic step and are responsible for its distinctive properties.

There is a considerable body of information showing that *T. gondii*-infected cells may become resistant to apoptotic stimuli, probably mediated via parasite-induced activation of NFκB and the transcriptional up-regulation of anti-apoptotic genes, as well as by inhibition of cytochrome C release and caspase-9 activation (reviewed in [49]). However several of these reports are studies on human cells which lack IFNγ-inducible IRG proteins [50] while those infecting mouse cells *in vitro* used the virulent RH strain [51]–[55], so the relevance to our own observations is not explicit. The necrotic death that we report with avirulent strains evidently overrides *T. gondii*-mediated control of apoptosis by superimposing a different lethal programme under the control of IFNγ. As noted above, however, it is unclear what role IFNγ plays in the necrotic programme. Is it simply the induction of IRG proteins leading to the disruption of the vacuole with release of an unknown pro-necrotic signal, or are there further IFNγ-induced components that play a crucial role in the subsequent necrotic events?

It is remarkable that the death of IFNγ-induced cells infected with avirulent *T. gondii* strains has not been noticed before since it is a large scale and relatively early event that, with reasonable MOIs, is immediately apparent after a cursory look down the microscope. There is, however, an isolated report of IFNγ-dependent cell death in a study on 3T3 fibroblasts infected with the close relative of *T. gondii*, *Neospora caninum* [56]. In this study the cell death observed was attributed to apoptosis, but this diagnosis was not confirmed experimentally. In the same study, in contrast, no cell death was detected in cells infected with *T. gondii*, encouraging the authors to suggest that the two related parasites stimulate different processes. In fact, however, they used virulent RH strain *T. gondii*, and as we show here and elsewhere [37], no necrotic death is associated with this strain because of its inhibition of IRG function.

The uracil incorporation assay for IFNγ-dependent inhibition of *T. gondii* replication is now 24 years old and has been used extensively in the *T. gondii* research field [14],[15]. It is an extremely useful and sensitive assay. However the necrotic death of many IFNγ-induced cells infected with *T. gondii* in the first 8 hours of the assay means that ^3^H-uracil added to the assay after 48 hours and measured at 72 hours to a certain extent records not only failure of the parasites to replicate, but also the fact that there are fewer live cells for the *T. gondii* to infect.

A further issue provoked by our results is their relevance to the *in vivo* situation. Although the process we describe leads to the death of individual ME49 *T. gondii* in infected, IFNγ-induced cells, additional parasites infecting the same cell seem to remain viable so long as the integrity of their vacuoles is maintained. In the Videos S9 and S10 a *T. gondii* enclosed in an intact vacuole that has accumulated no Irga6 remains visible until the last frame. As the host cell dies by necrosis following the earlier disruption of another vacuole this surviving parasite can be seen in the phase contrast image apparently to escape from the corpse of its host by active movement. The vision is fleeting, but a recent publication documented large-scale egress of live *T. gondii* from IFNγ-induced astrocytes [16] and egress has been reported before [57]–[59]. We did not see exit on such a scale in our experiments, but it may nevertheless be questioned whether the death of individual intracellular *T. gondii* documented here is the major adaptive purpose of the necrotic process. Melzer *et al* assert that the putatively released *T. gondii* in the astrocyte system are no longer capable of invasion, which would also obviously contribute to an adaptive advantage [16]. A further plausible and not exclusive hypothesis is that the local release of pro-inflammatory signals such as HMGB1 from necrotic cells, favouring local accumulation of cellular components of the innate and adaptive immune systems, contributes significantly to the resistance process *in vivo* [22].

An additional reason for seeing the process we describe as important for *in vivo* events in *T. gondii* infection is the correlation between the induction of necrotic death in cellular infection *in vitro* and the genetics of *T. gondii* virulence defined by lethality in mice. For type I strains, high lethality *in vivo* is associated with reduced IRG protein accumulation on the PVM (9% RH vacuoles are Irgb6-positive compared with 70% ME49 vacuoles [37]), no vacuole rupture and no subsequent necrotic death of infected cells *in vitro*. Several virulence factors have now been identified in rhoptry secretions [41]–[43] and it is plausible that one or more of these is dedicated to interference with the IRG resistance mechanism, presumably at the vacuolar loading step. The vacuolar loading step appears to be the Achilles heel of the IRG resistance mechanism since it was reported recently that failure of IFNγ-inducible resistance to *Chlamydia muridarum* in mice is also associated with failure of IRG proteins to accumulate normally on the chlamydial inclusions, compared with successful IRG loading and efficient IRG-mediated resistance to the closely-related *C. trachomatis* [60]. In this case, however, resistance to the IRG system was functionally dominant, since the presence of *C. muridarum* in the cell actively inhibited the accumulation of IRG protein on co-infecting *C. trachomatis*. The molecular basis of the attack of *C. muridarum* on the IRG system must therefore be different from the attack by virulent *T. gondii*, an unsurprising conclusion in view of the taxonomic disparity between these two pathogens. It was recently shown that several of the IFNγ-inducible p65 guanylate binding proteins also accumulate on the *T. gondii* PVM, and this accumulation too failed with a virulent strain [46]. Does this imply yet another defence mechanism? In the absence of new information it is difficult to see the defeat of innate resistance mechanisms in mice by virulent *T. gondii* as a success for either partner. The mouse is destined to die within 10 days [1],[2], hardly a triumph for the mouse, while death of the mouse before large-scale bradyzoite transition and encystment reduces the chance for *T. gondii* to infect any cat lucky enough to catch the mouse in the short time window before it dies. The adaptive significance of the virulent phenotype may be more relevant to the genotype of a different intermediate host, for example the rat, *Rattus norvegicus*, which is not vulnerable to virulent strains of *T. gondii* [61].

The IRG system is a complex resistance mechanism with multiple interacting components and many unexplained features. Its adaptive significance for pathogen resistance in mice is hard to judge at present since only *C. muridarum* and *T. gondii* of the organisms studied are natural mouse pathogens [62]. From data presently available, however, it seems likely that the IRG system is under selective pressure from polymorphic pathogens in the natural environment and may display significant variation as a consequence. This aspect of the IRG system has not yet been investigated systematically. What is, however, clear, is that IFNγ-inducible IRG genes are not present in humans [50], leaving resistance to pathogens such as *T. gondii* and *Chlamydia* to be organised by other means. At present the principal candidate mechanism in this role for *T. gondii* is the IFNγ-inducible catabolic enzyme, indoleamine dioxygenase, whose expression results in depletion of free cytosolic tryptophan [63]. It will, however, be of some interest to monitor the fate of *T. gondii* in IFNγ-induced human cells at the same level of resolution as has been employed here and elsewhere in mouse cells.

## Materials and Methods

### Expression constructs

The Irga6-ctag1-EGFP construct was employed for live-imaging experiments following the demonstration (Sascha Martens, unpublished) that this doubly tagged construct showed normal localisation behaviour following transfection into IFNγ-induced cells. Irga6 carrying a single C-terminal EGFP tag formed aggregates in cells and probably activates spontaneously [20],[40]. The construct was generated by amplification of the Irga6ctag1 sequence from pGW1H-Irga6ctag1 [40] using Irga6ctag1 forward 5′-cccccccccgtcgaccaccatgggtcagctgttctcttcacctaag-3′ and reverse 5′-cccccccccgtcgacgtcacgatgcggccgctcgagtcggcctag-3′ primers and cloned into pEGFP-N3 (Clontech) by SalI digestion. The pmDsRed-N3 and pmCherry-N3 constructs were generated by amplification of mDsRed and mCherry respectively from pDsRed-Monomer-N In-Fusion (Clontech) or mCherry-pRsetB (generous gift from Dr.. Roger Y. Tsien, UCSD) using the following primers, and inserted into pEGFP-N3 following BamHI/NotI digestion: pmDsRed-N3 forward 5′- cccccccccggatccatggacaacaccgaggacgtcat-3′ and reverse 5′-cccccccccgcggccgcctactgggagccggagtggcgggc-3′, pCherry-N3 forward 5′-cccccccccggatccatggtgagcaagggcgaggagga-3′ and reverse 5′-cccccccccgcggccgcctacttgtacagctcgtccatgc-3′. EGFP-LC3, pGW1H-Irgb6-FLAG, pGW1H-Irgb6-K69A-FLAG constructs were generated as described [17],[20],[40].

### Cell culture, *Toxoplasma gondii* strains and infection of cells *in vitro*

C57BL/6 embryonic fibroblasts (MEFs) were prepared from mice at day 14 post coitum and cultured in DMEM (high glucose) (Invitrogen) supplemented with 10% FCS (Biochrom AG, Berlin, Germany), 2 mM L-glutamine, 1 mM sodium pyruvate, non-essential amino acids, 100 U/ml penicillin, 100 mg/ml streptomycin (all PAA, Pasching, Austria) as described [9]. C57BL/6 bone-marrow derived macrophages (BMMs) were prepared from young adult mouse bone marrow cells cultured in DMEM (high glucose) containing 10% L929 P2 cell-conditioned medium and supplemented with 10% FCS (Biochrom AG, Berlin, Germany), 2 mM L-glutamine, 1 mM sodium pyruvate, non-essential amino acids, 100 U/ml penicillin, 100 mg/ml streptomycin. Apoptosis was induced by adding TNFα (40 ng/ml, PeproTech, NJ, USA) and cycloheximide (10 µg/ml, Sigma-Aldrich) to the medium. Inflammasome activation is induced by incubating cells with LPS (1 µg/µl, Sigma-Aldrich) for 24 hours, and then treated with nigericin (20 µM, Sigma-Aldrich).

*T. gondii* tachyzoites from the type I strain RH-YFP or type II strain ME49 were maintained by serial passage in confluent monolayers of human foreskin fibroblasts (HS27, ATCC CRL-1634) as described [17]. RH-YFP parasites were propagated in the presence of Chloramphenicol (3.2 µg/ml, Sigma-Aldrich) to maintain the stably integrated YFP expression plasmid containing a chloramphenicol acetyltransferase selection marker [64]. Both *T. gondii* strains were a generous gift from Dr. Gaby Reichmann, Medical Microbiology, University of Düsseldorf.

Cells were transiently transfected using FuGENE6 (Roche) according to the manufacturer's instructions, and induced with mouse IFNγ (PeproTech, NJ, USA) 24 hours before infection with *T. gondii* tachyzoites at the MOI indicated in the data element.

### Live-cell imaging

All the live cell imaging experiments were performed in μ-slide I chambers (Ibidi, Munich)., which are exceptionally well-suited to this kind of experimentation. For live cell experiments, all procedures including transfection, medium modification (addition of IFNγ etc) and infection with *T. gondii* could be carried out while the cells were continuously incubated in an observation volume of 100 µL and cells could be maintained in excellent condition for at least 20 hours. Cells were incubated at 37°C in phenol-red-free DMEM supplemented with 10% FCS, 20 mM HEPES pH 7.4, 2 mM L-glutamine, 1 mM sodium pyruvate, 1× non-essential amino acids, 100 U/ml penicillin, and 100 µg/ml streptomycin. MEFs were transfected and stimulated with 200 U/ml IFNγ for 24 hours. After infection with *T. gondii*, the cells were observed with a Zeiss Axiovert 200 M motorized microscope fitted with a wrap-around temperature-controlled chamber, using an EC “Plan-Neofluar” 40×/1.30 Oil Ph3 objective (Zeiss). The time-lapse images were obtained and processed by Axiovision 4.6 software (Zeiss). Phosphatidylserine was detected by adding 1% (v/v) Alexa-555 labeled annexin V (Molecular Probes, Invitrogen) with 2.5 mM CaCl_2_ into the incubation medium.

### Immunological reagents

The following serological reagents were used for immunofluorescence (IF) and western blot (WB): anti-Irga6 rabbit antiserum 165 (IF 1∶8000, [17]), anti-Irgb6 goat antiserum A20 (IF 1∶100, Santa Cruz Biotechnology), anti-GRA7 mouse monoclonal antibody (IF 1∶1000, gift from Dr. Gaby Reichmann, Medical Microbiology, University of Düsseldorf. [17]), anti-FLAG M2 mouse monoclonal antibody (IF 1∶4000, Sigma-Aldrich), anti-cytochrome C mouse monoclonal antibody (IF 1∶1000, BD PharMingen Clone: 6H2.B4), anti-LAMP1 rat monoclonal antibody 1D4B (IF 1∶1000, University of Iowa, USA), anti-PARP rabbit polyclonal antibody (WB 1∶1000, Cell Signaling Technology, MA, USA), anti-cleaved caspase-3 rabbit polyclonal antibody (WB 1∶1000, Cell signaling Technology, MA, USA), anti-HMGB-1 rabbit polyclonal antibody (WB 1∶250, Abcam), anti-Calnexin SPA-865 rabbit antiserum (WB 1∶10000, Stressgen), anti-IL-1β rabbit polyclonal antibody (WB 1∶2500, Abcam), anti-caspase-1 p10 M20 goat polyclonal antibody (WB 1∶200, Santa Cruz Biotechnology), goat anti-mouse Alexa 488 and 546, goat anti-rabbit Alexa 488 and 546, donkey anti-rat Alexa 488, donkey anti-goat Alexa 350, 488, 546 and 647, donkey anti-mouse Alexa 488, 555 and 647, donkey anti-rabbit Alexa 488, 555 and 647 (IF 1∶1000, Molecular Probes, Invitrogen), donkey anti-rabbit HRP (Amersham), donkey anti-goat HRP (Santa Cruz Biotechnology) and goat anti-mouse HRP (Pierce Biotechnology) (WB 1∶5000).

### Immunofluorescent staining

Immunofluorescent staining was performed on paraformaldehyde-fixed cells essentially as described earlier [17]. Images were taken with a Zeiss Axioplan II fluorescence microscope equipped with an AxioCam MRm camera (Zeiss) and processed with Axiovision 4.6 software (Zeiss). 4′,6-Diamidine-2′-phenylindole dihydrochloride (DAPI, Invitrogen) was used for nuclear counterstaining at a final concentration of 0.5 µg/ml. Intracellular parasites were identified by immunostaining for vacuolar localisation of the *T. gondii* protein dense granule proteins, GRA7 or by their distinctive appearance in phase contrast.

### Cell viability assay

MEFs (7500 cells/well) or BMMs (2×10^4^ cells/well) were seeded into 96-well plates and treated with IFNγ or under control conditions for 24 hours. The cells were then infected with *T. gondii* at the indicated MOI for 8 hours. Thereafter, viable cells were quantified by the CellTiter 96 AQueous non-radioactive cell proliferation assay (Promega) according to the manufacturer's instructions. The absorption of the bio-reduced form (formazan) of a substrate (MTS) generated by metabolically active cells during incubation at 37°C for 2–4 hours was measured in an ELISA reader (Molecular Devices) at 490 nm. The quantity of formazan product is proportional to the number of living cells in the culture.

## Supporting Information

Figure S1 IFNγ-dependent host cell death upon *T. gondii* avirulent strain infection. MEFs were induced with 200 U/ml IFNγ or left untreated for 24 hours, and then infected with *T. gondii* ME49 strain at a MOI of 5. At 2 hours after infection, debris and extracellular *T. gondii* were washed off. 6 hours later, propidium iodide (0.4 µg/ml, final concentration) was added without further treatment before imaging by inverted microscopy at 10× objective. Scale bar 200 µm.
(9.37 MB TIF)

Figure S2 Live *T. gondii* PVs do not fuse with lysosomes. MEFs were treated with 200 U/ml IFNγ for 24 hours and then infected with live (upper panel) or heat-killed (lower panel) ME49 *T. gondii* for 4 hours. Cells were fixed and stained for LAMP1 (red). Arrows indicated the intracellular *T. gondii*. Scale bar 10 µm.
(5.56 MB TIF)

Table S1 Time-course of the IRG protein induced IFNγ-dependent necrotic programme. Table S1 gives the times in minutes between different steps in the necrotic programme as observed by time-lapse microscopy.
(0.05 MB PDF)

Video S1 Fig1A Disruption of Irga6-positive *T. gondii* PVM. IFNγ-treated MEFs were transfected with Irga6-ctag1-EGFP and infected with ME49 *T. gondii*. Time-lapse video was started 1 hour after infection and Irga6-ctag1-EGFP images were collected every 1 minute. The video is presented at 2 frames per second. Scale bar: 5 µm.
(0.14 MB MOV)

Video S2 Fig1B Disruption of Irga6-positive *T. gondii* PVM. IFNγ-treated MEFs were transfected with Irga6-ctag1-EGFP and infected with ME49 *T. gondii*. Time-lapse video was started 1 hour after infection and Irga6-ctag1-EGFP images were collected every 1 minute. The video is presented at 2 frames per second. Scale bar: 5 µm.
(0.08 MB MOV)

Video S3 Fig2A_EGFP *T. gondii* is killed in the cytosol in IFNγ-treated cells. IFNγ-treated MEFs were transfected with pEGFP-N3. Time-lapse video was started 2 hours after infection with ME49 *T. gondii*, and images were collected every 5 minutes. The video is presented at 2 frames per second. Scale bar: 5 µm.
(0.12 MB MOV)

Video S4 Fig2A_Phc *T. gondii* is killed in the cytosol in IFNγ-treated cells. IFNγ-treated MEFs were transfected with pEGFP-N3. Time-lapse video was started 2 hours after infection with ME49 *T. gondii*, and images were collected every 5 minutes. The video is presented at 2 frames per second. Scale bar: 5 µm.
(0.30 MB MOV)

Video S5 Fig1C2B_Irga6-ctag1-EGFP *T. gondii* is permeablised in the host cytosol after the disruption of PVM. IFNγ-treated MEFs were cotransfected with Irga6-ctag1-EGFP and pCherry. Time-lapse video was started 1 hour after infection with ME49 *T. gondii*, and images were collected every 3 minutes. The video is presented at 2 frames per second. Scale bar: 5 µm.
(0.04 MB MOV)

Video S6 Fig1C2B_Phc *T. gondii* is permeablised in the host cytosol after the disruption of PVM. IFNγ-treated MEFs were cotransfected with Irga6-ctag1-EGFP and pCherry. Time-lapse video was started 1 hour after infection with ME49 *T. gondii*, and images were collected every 3 minutes. The video is presented at 2 frames per second. Scale bar: 5 µm.
(0.15 MB MOV)

Video S7 Fig1C2B_Cherry *T. gondii* is permeablised in the host cytosol after the disruption of PVM. IFNγ-treated MEFs were cotransfected with Irga6-ctag1-EGFP and pCherry. Time-lapse video was started 1 hour after infection with ME49 *T. gondii*, and images were collected every 3 minutes. The video is presented at 2 frames per second. Scale bar: 5 µm.
(0.05 MB MOV)

Video S8 Fig1C2B_Merge *T. gondii* is permeablised in the host cytosol after the disruption of PVM. IFNγ-treated MEFs were cotransfected with Irga6-ctag1-EGFP and pCherry. Time-lapse video was started 1 hour after infection with ME49 *T. gondii*, and images were collected every 3 minutes. The video is presented at 2 frames per second. Scale bar: 5 µm.
(0.07 MB MOV)

Video S9 Fig4_Merge Host cells die after disruption of the PVM and death of the *T. gondii*. IFNγ-treated MEFs were cotransfected with Irga6-ctag1-EGFP and pmDsRed. Time-lapse video was started 1 hour after infection with ME49 *T. gondii*, and images were collected every 5 minutes. The video is presented at 2 frames per second. Scale bar: 10 µm.
(0.52 MB MOV)

Video S10 Fig4_Phc Host cells die after disruption of the PVM and death of the *T. gondii*. IFNγ-treated MEFs were cotransfected with Irga6-ctag1-EGFP and pmDsRed. Time-lapse video was started 1 hour after infection with ME49 *T. gondii*, and images were collected every 5 minutes. The video is presented at 2 frames per second. Scale bar: 10 µm.
(1.30 MB MOV)

Video S11 Fig5A_Merge IFNγ-treated *T. gondii* infected host cells undergo necrosis. IFNγ-treated MEFs were transfected with pEGFP-N3. Time-lapse video was started 1 hour after infection with ME49 *T. gondii*, and images were collected every 5 minutes. Phosphatidylserine were detected by adding 1% (v/v) Alexa-555-labeled annexin V with 2.5 mM CaCl_2_ into the medium. The video is presented at 2 frames per second. Scale bar: 10 µm.
(0.23 MB MOV)

Video S12 Fig5A_Phc IFNγ-treated *T. gondii* infected host cells undergo necrosis. IFNγ-treated MEFs were transfected with pEGFP-N3. Time-lapse video was started 1 hour after infection with ME49 *T. gondii*, and images were collected every 5 minutes. Phosphatidylserine were detected by adding 1% (v/v) Alexa-555-labeled annexin V with 2.5 mM CaCl_2_ into the medium. The video is presented at 2 frames per second. Scale bar: 10 µm.
(0.89 MB MOV)

Video S13 Fig6B_EGFP-LC3 Live imaging of EGFP-LC3 in IFNγ-treated *T. gondii* infected cells. IFNγ-treated MEFs were transfected with EGFP-LC3. Time-lapse video was started 2 hour after infection with ME49 *T. gondii*, and images were collected every 5 minutes. The video is presented at 2 frames per second. Scale bar: 10 µm.
(0.36 MB MOV)

Video S14 Fig6B_Phc Live imaging of EGFP-LC3 in IFNγ-treated *T. gondii* infected cells. IFNγ-treated MEFs were transfected with EGFP-LC3. Time-lapse video was started 2 hour after infection with ME49 *T. gondii*, and images were collected every 5 minutes. The video is presented at 2 frames per second. Scale bar: 10 µm.
(0.80 MB MOV)

## References

[1] Saeij JP, Boyle JP, Boothroyd JC (2005). Differences among the three major strains of Toxoplasma gondii and their specific interactions with the infected host.. Trends Parasitol.

[2] Sibley LD, Boothroyd JC (1992). Virulent strains of Toxoplasma gondii comprise a single clonal lineage.. Nature.

[3] Frenkel JK (1988). Pathophysiology of toxoplasmosis.. Parasitol Today.

[4] Hunter CA, Remington JS (1994). Immunopathogenesis of toxoplasmic encephalitis.. J Infect Dis.

[5] Yap GS, Sher A (1999). Cell-mediated immunity to Toxoplasma gondii: initiation, regulation and effector function.. Immunobiology.

[6] Scharton-Kersten TM, Wynn TA, Denkers EY, Bala S, Grunvald E (1996). In the absence of endogenous IFN-gamma, mice develop unimpaired IL-12 responses to Toxoplasma gondii while failing to control acute infection.. J Immunol.

[7] Scharton-Kersten TM, Yap G, Magram J, Sher A (1997). Inducible nitric oxide is essential for host control of persistent but not acute infection with the intracellular pathogen Toxoplasma gondii.. J Exp Med.

[8] Yap GS, Ortmann R, Shevach E, Sher A (2001). A heritable defect in IL-12 signaling in B10.Q/J mice. II. Effect on acute resistance to Toxoplasma gondii and rescue by IL-18 treatment.. J Immunol.

[9] Boehm U, Guethlein L, Klamp T, Ozbek K, Schaub A (1998). Two families of GTPases dominate the complex cellular response to interferon-g.. J Immunol.

[10] Halonen SK, Taylor GA, Weiss LM (2001). Gamma Interferon-Induced Inhibition of Toxoplasma gondii in Astrocytes Is Mediated by IGTP.. Infection and Immunity.

[11] Taylor GA, Collazo CM, Yap GS, Nguyen K, Gregorio TA (2000). Pathogen-specific loss of host resistance in mice lacking the IFN-gamma-inducible gene IGTP.. Proc Natl Acad Sci U S A.

[12] Collazo CM, Yap GS, Sempowski GD, Lusby KC, Tessarollo L (2001). Inactivation of LRG-47 and IRG-47 Reveals a Family of Interferon {gamma}-inducible Genes with Essential, Pathogen-specific Roles in Resistance to Infection.. J Exp Med.

[13] Könen-Waisman S, Howard JC (2007). Cell-autonomous immunity to Toxoplasma gondii in mouse and man.. Microbes Infect.

[14] Pfefferkorn ER, Guyre PM (1984). Inhibition of growth of Toxoplasma gondii in cultured fibroblasts by human recombinant gamma interferon.. Infect Immun.

[15] Pfefferkorn ER (1984). Interferon gamma blocks the growth of Toxoplasma gondii in human fibroblasts by inducing the host cells to degrade tryptophan.. Proc Natl Acad Sci U S A.

[16] Melzer T, Duffy A, Weiss LM, Halonen SK (2008). IGTP is necessary for Toxoplasma Vacuolar Disruption and Induces Parasite Egression in IFN{gamma} Stimulated Astrocytes.. Infect Immun.

[17] Martens S, Parvanova I, Zerrahn J, Griffiths G, Schell G (2005). Disruption of Toxoplasma gondii parasitophorous vacuoles by the mouse p47 resistance GTPases.. PLoS Pathog.

[18] Butcher BA, Greene RI, Henry SC, Annecharico KL, Weinberg JB (2005). p47 GTPases Regulate Toxoplasma gondii Survival in Activated Macrophages.. Infect Immun.

[19] Ling YM, Shaw MH, Ayala C, Coppens I, Taylor GA (2006). Vacuolar and plasma membrane stripping and autophagic elimination of Toxoplasma gondii in primed effector macrophages.. J Exp Med.

[20] Hunn JP, Koenen-Waisman S, Papic N, Schroeder N, Pawlowski N (2008). Regulatory interactions between IRG resistance GTPases in the cellular response to Toxoplasma gondii.. Embo J.

[21] Zhao ZJ, Fux B, Goodwin M, DUnay IR, Strong D (2008). ATG5 is essential for cellular immunity in vivo and recruitment of a p47 GTPase to the Toxoplasma gondii parasitophorous vacuole in activated macrophages.. Cell Host Microbe.

[22] Scaffidi P, Misteli T, Bianchi ME (2002). Release of chromatin protein HMGB1 by necrotic cells triggers inflammation.. Nature.

[23] Edinger AL, Thompson CB (2004). Death by design: apoptosis, necrosis and autophagy.. Curr Opin Cell Biol.

[24] Vermes I, Haanen C, Steffens-Nakken H, Reutelingsperger C (1995). A novel assay for apoptosis. Flow cytometric detection of phosphatidylserine expression on early apoptotic cells using fluorescein labelled Annexin V.. J Immunol Methods.

[25] Goldstein JC, Waterhouse NJ, Juin P, Evan GI, Green DR (2000). The coordinate release of cytochrome c during apoptosis is rapid, complete and kinetically invariant.. Nat Cell Biol.

[26] Tewari M, Quan LT, O'Rourke K, Desnoyers S, Zeng Z (1995). Yama/CPP32 beta, a mammalian homolog of CED-3, is a CrmA-inhibitable protease that cleaves the death substrate poly(ADP-ribose) polymerase.. Cell.

[27] Brennan MA, Cookson BT (2000). Salmonella induces macrophage death by caspase-1-dependent necrosis.. Mol Microbiol.

[28] Cervantes J, Nagata T, Uchijima M, Shibata K, Koide Y (2008). Intracytosolic Listeria monocytogenes induces cell death through caspase-1 activation in murine macrophages.. Cell Microbiol.

[29] Schroeder GN, Jann NJ, Hilbi H (2007). Intracellular type III secretion by cytoplasmic Shigella flexneri promotes caspase-1-dependent macrophage cell death.. Microbiology.

[30] Yu HB, Finlay BB (2008). The caspase-1 inflammasome: a pilot of innate immune responses.. Cell Host Microbe.

[31] Hentze H, Lin XY, Choi MS, Porter AG (2003). Critical role for cathepsin B in mediating caspase-1-dependent interleukin-18 maturation and caspase-1-independent necrosis triggered by the microbial toxin nigericin.. Cell Death Differ.

[32] Cheneval D, Ramage P, Kastelic T, Szelestenyi T, Niggli H (1998). Increased mature interleukin-1beta (IL-1beta) secretion from THP-1 cells induced by nigericin is a result of activation of p45 IL-1beta-converting enzyme processing.. J Biol Chem.

[33] Willingham SB, Bergstralh DT, O'Connor W, Morrison AC, Taxman DJ (2007). Microbial pathogen-induced necrotic cell death mediated by the inflammasome components CIAS1/cryopyrin/NLRP3 and ASC.. Cell Host Microbe.

[34] Levine B (2005). Eating oneself and uninvited guests; autophagy-related pathways in cellular defense.. Cell.

[35] Levine B, Deretic V (2007). Unveiling the roles of autophagy in innate and adaptive immunity.. Nat Rev Immunol.

[36] Schmid D, Munz C (2007). Innate and adaptive immunity through autophagy.. Immunity.

[37] Zhao YO, Rohde C, Lilue JT, Könen-Waisman S, Khaminets A (2009). Toxoplasma gondii and the IRG resistance system in mice.. Mem Inst Oswaldo Cruz.

[38] Singh SB, Davis AS, Taylor GA, Deretic V (2006). Human IRGM Induces Autophagy to Eliminate Intracellular Mycobacteria.. Science.

[39] Gutierrez MG, Master SS, Singh SB, Taylor GA, Colombo MI (2004). Autophagy is a defense mechanism inhibiting BCG and Mycobacterium tuberculosis survival in infected macrophages.. Cell.

[40] Papic N, Hunn JP, Pawlowski N, Zerrahn J, Howard JC (2008). Inactive and active states of the interferon-inducible resistance GTPase, Irga6, In Vivo.. J Biol Chem.

[41] Saeij JP, Boyle JP, Coller S, Taylor S, Sibley LD (2006). Polymorphic secreted kinases are key virulence factors in toxoplasmosis.. Science.

[42] Saeij JP, Coller S, Boyle JP, Jerome ME, White MW (2007). Toxoplasma co-opts host gene expression by injection of a polymorphic kinase homologue.. Nature.

[43] Taylor S, Barragan A, Su C, Fux B, Fentress SJ (2006). A secreted serine-threonine kinase determines virulence in the eukaryotic pathogen Toxoplasma gondii.. Science.

[44] Praefcke GJK, McMahon HT (2004). The dynamin superfamily: universal membrane tubulation and fission molecules?. Nature Reviews Molecular Cell Biology.

[45] Martens S, Howard J (2006). The Interferon-Inducible GTPases.. Annu Rev Cell Dev Biol.

[46] Degrandi D, Konermann C, Beuter-Gunia C, Kresse A, Wurthner J (2007). Extensive characterization of IFN-induced GTPases mGBP1 to mGBP10 involved in host defense.. J Immunol.

[47] Halonen SK, Weiss LM (2000). Investigation into the mechanism of gamma interferon-mediated inhibition of Toxoplasma gondii in murine astrocytes.. Infect Immun.

[48] Hitziger N, Dellacasa I, Albiger B, Barragan A (2005). Dissemination of Toxoplasma gondii to immunoprivileged organs and role of Toll/interleukin-1 receptor signalling for host resistance assessed by in vivo bioluminescence imaging.. Cell Microbiol.

[49] Carmen JC, Sinai AP (2007). Suicide prevention: disruption of apoptotic pathways by protozoan parasites.. Mol Microbiol.

[50] Bekpen C, Hunn JP, Rohde C, Parvanova I, Guethlein L (2005). The interferon-inducible p47 (IRG) GTPases in vertebrates: loss of the cell autonomous resistance mechanism in the human lineage.. Genome Biol.

[51] Butcher BA, Kim L, Johnson PF, Denkers EY (2001). Toxoplasma gondii tachyzoites inhibit proinflammatory cytokine induction in infected macrophages by preventing nuclear translocation of the transcription factor NF-kappa B.. J Immunol.

[52] Nash PB, Purner MB, Leon RP, Clarke P, Duke RC (1998). Toxoplasma gondii-infected cells are resistant to multiple inducers of apoptosis.. J Immunol.

[53] Payne TM, Molestina RE, Sinai AP (2003). Inhibition of caspase activation and a requirement for NF-kappaB function in the Toxoplasma gondii-mediated blockade of host apoptosis.. J Cell Sci.

[54] Molestina RE, Payne TM, Coppens I, Sinai AP (2003). Activation of NF-kappaB by Toxoplasma gondii correlates with increased expression of antiapoptotic genes and localization of phosphorylated IkappaB to the parasitophorous vacuole membrane.. J Cell Sci.

[55] Carmen JC, Hardi L, Sinai AP (2006). Toxoplasma gondii inhibits ultraviolet light-induced apoptosis through multiple interactions with the mitochondrion-dependent programmed cell death pathway.. Cell Microbiol.

[56] Nishikawa Y, Makala L, Otsuka H, Mikami T, Nagasawa H (2002). Mechanisms of apoptosis in murine fibroblasts by two intracellular protozoan parasites, Toxoplasma gondii and Neospora caninum.. Parasite Immunol.

[57] Caldas LA, de Souza W, Attias M (2007). Calcium ionophore-induced egress of Toxoplasma gondii shortly after host cell invasion.. Vet Parasitol.

[58] Persson EK, Agnarson AM, Lambert H, Hitziger N, Yagita H (2007). Death receptor ligation or exposure to perforin trigger rapid egress of the intracellular parasite Toxoplasma gondii.. J Immunol.

[59] Moudy R, Manning TJ, Beckers CJ (2001). The loss of cytoplasmic potassium upon host cell breakdown triggers egress of Toxoplasma gondii.. J Biol Chem.

[60] Coers J, Bernstein-Hanley I, Grotsky D, Parvanova I, Howard JC (2008). Chlamydia muridarum evades growth restriction by the IFN-gamma-inducible host resistance factor Irgb10.. J Immunol.

[61] Dubey JP, Shen SK, Kwok OC, Frenkel JK (1999). Infection and immunity with the RH strain of Toxoplasma gondii in rats and mice.. J Parasitol.

[62] Coers J, Starnbach MN, Howard JC (2009). Modeling infectious disease in mice: co-adaptation and the role of host-specific IFNγ responses.. PLoS Pathog.

[63] MacKenzie CR, Heseler K, Muller A, Daubener W (2007). Role of indoleamine 2,3-dioxygenase in antimicrobial defence and immuno-regulation: tryptophan depletion versus production of toxic kynurenines.. Curr Drug Metab.

[64] Gubbels MJ, Li C, Striepen B (2003). High-throughput growth assay for Toxoplasma gondii using yellow fluorescent protein.. Antimicrob Agents Chemother.

