# Supplementary figures and images for: Disruption of the Toxoplasma gondii Parasitophorous Vacuole by IFNγ-Inducible Immunity-Related GTPases (IRG Proteins) Triggers Necrotic Cell Death
Source: PLoS Pathog. 2009 Feb 6;5(2):e1000288. doi: 10.1371/journal.ppat.1000288 (PMC2629126; doi:10.1371/journal.ppat.1000288)

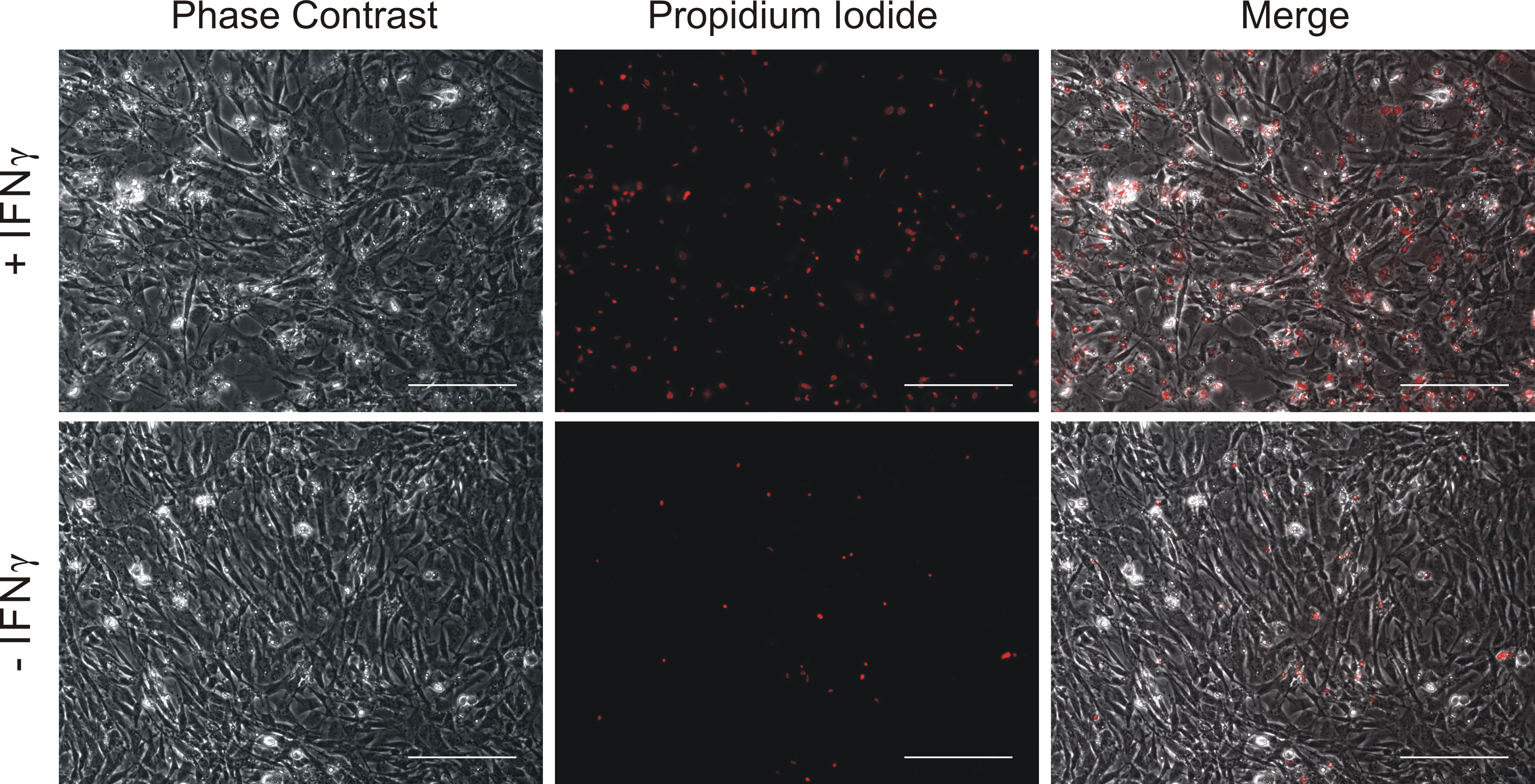

Supplement: Figure S1 — IFNγ-dependent host cell death upon T. gondii avirulent strain infection. MEFs were induced with 200 U/ml IFNγ or left untreated for 24 hours, and then infected with T. gondii ME49 strain at a MOI of 5. At 2 hours after infection, debris and extracellular T. gondii were washed off. 6 hours later, propidium iodide (0.4 µg/ml, final concentration) was added without further treatment before imaging by inverted microscopy at 10× objective. Scale bar 200 µm. (9.37 MB TIF) [file ppat.1000288.s001.tif]

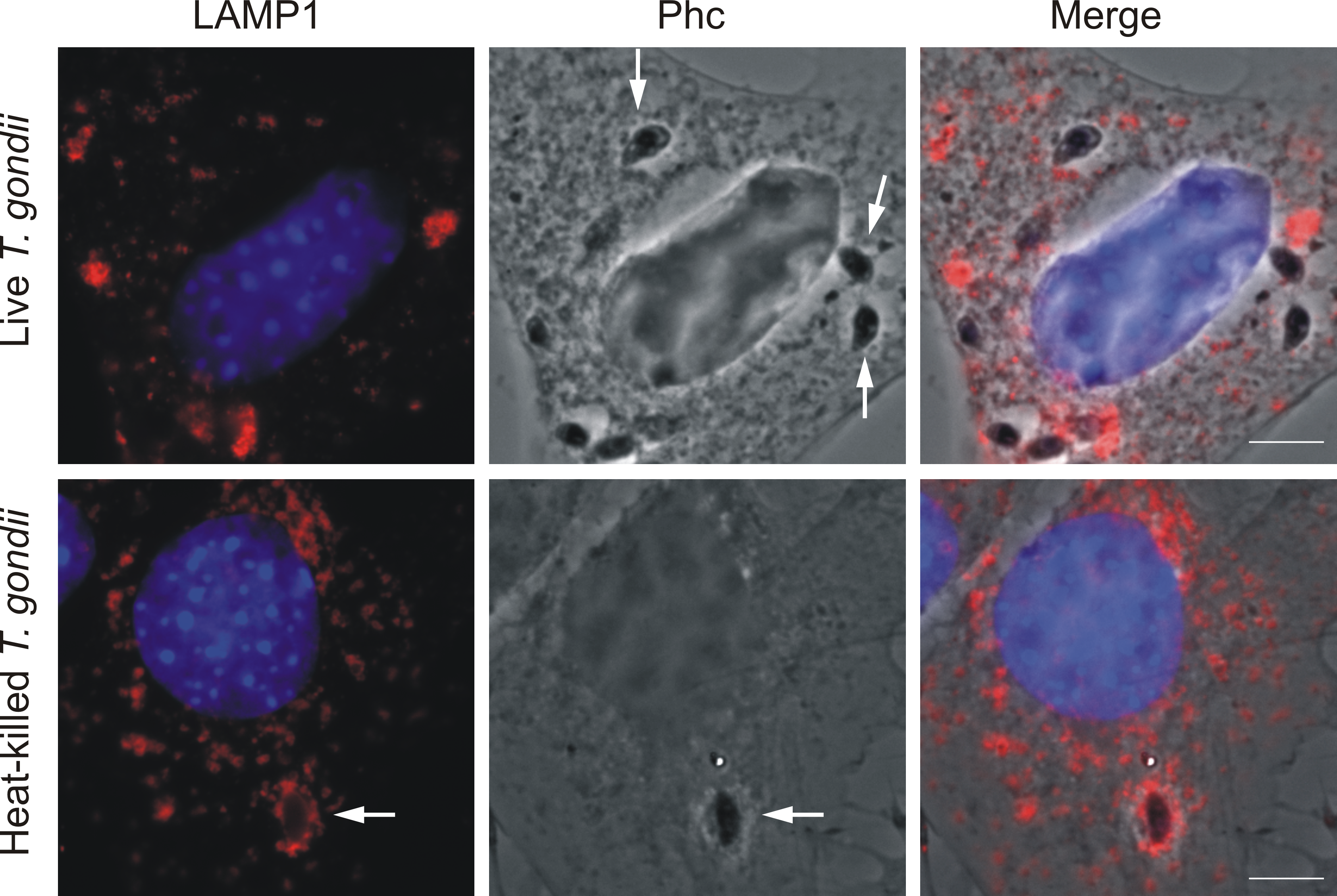

Supplement: Figure S2 — Live T. gondii PVs do not fuse with lysosomes. MEFs were treated with 200 U/ml IFNγ for 24 hours and then infected with live (upper panel) or heat-killed (lower panel) ME49 T. gondii for 4 hours. Cells were fixed and stained for LAMP1 (red). Arrows indicated the intracellular T. gondii. Scale bar 10 µm. (5.56 MB TIF) [file ppat.1000288.s002.tif]
