# Supplementary material for: Disruption of the Toxoplasma gondii Parasitophorous Vacuole by IFNγ-Inducible Immunity-Related GTPases (IRG Proteins) Triggers Necrotic Cell Death
Source: PLoS Pathog. 2009 Feb 6;5(2):e1000288. doi: 10.1371/journal.ppat.1000288 (PMC2629126; doi:10.1371/journal.ppat.1000288)
Supplement: Table S1 — Time-course of the IRG protein induced IFNγ-dependent necrotic programme. Table S1 gives the times in minutes between different steps in the necrotic programme as observed by time-lapse microscopy. (0.05 MB PDF) [file ppat.1000288.s003.pdf]

Table S1 Time-course of the IRG protein induced IFNγ-dependent necrotic programme.

Time from infection[1] to PV disruption[2]	PV disruption to *T. g.* permeabilisation[3]	*T. g.* permeabilisation to cell necrosis[4]	PV disruption to cell necrosis	Time from infection to cell necrosis
135			280	415
135			85	220
160			20	180
				130[5]
96			24	120
				260[5]
170			25	195
		85		265
		30		110
		105		280
		80		225
70[6]	20	60	80	150[6]
	45[6]	5		150[6]
44	18	26	44	88
170	15	5	20	190
255	18	45	63	328
36	15	30	45	81
140	15	105	120	260
69[7]	27			
123(36-255) N=12	22(15-45) N=8	57(5-105) N=10	73(20-280) N=11	206(81-415) N=17

Table S1 gives the times in minutes between different steps in the necrotic programme as observed by time-lapse microscopy. Each line refers to an observation made on a single vacuole in IFNγ-induced MEFs infected with avirulent ME49 strain *T. gondii*. Incomplete information is due to use of different markers. Thus use of a cytosolic marker (EGFP or mDsRed) can inform about permeabilisation of the *T. gondii* and about breakdown of host cell plasma membrane integrity, but not about the disruption of the vacuolar membrane. Cell death was recorded either as loss of cytosolic markers or as the sudden morphological transition apparent from the phase contrast series. The last entry in each column gives the arithmetic mean and minimum/maximum time recorded between different steps. With the exception of one case at 45 minutes (footnote 6), the time between disruption and permeabilisation was the most consistent at close to 20 minutes.

Footnotes:
1. Time (minutes) from adding *T. gondii* to the cells to PV disruption (see next footnote).
2. PV disruption counted as time of breakdown of Irga6-ctag1-EGFP ring fluorescence.
3. Permeabilisation of *T. gondii* counted as time of entry of fluorescent cytosolic protein
4. Cell necrosis counted as time of loss of fluorescent cytosolic protein.
5. In these experiments imaging began after the PV disrupted.
6. Two vacuoles were observed to disrupt sequentially in this cell, the first *T. gondii* permeabilised after 20 minutes, the second after 45 minutes.
7. Imaging stopped before host cell necrosis.
